# Structure and mechanisms of transport of human Asc1/CD98hc amino acid transporter

**DOI:** 10.1038/s41467-024-47385-3

**Published:** 2024-04-06

**Authors:** Josep Rullo-Tubau, Maria Martinez-Molledo, Paola Bartoccioni, Ignasi Puch-Giner, Ángela Arias, Suwipa Saen-Oon, Camille Stephan-Otto Attolini, Rafael Artuch, Lucía Díaz, Víctor Guallar, Ekaitz Errasti-Murugarren, Manuel Palacín, Oscar Llorca

**Affiliations:** 1 Institute for Research in Biomedicine (IRB Barcelona), The Barcelona Institute of Science and Technology (BIST), Baldiri Reixac 10, E-08028 Barcelona, Spain; 2 Structural Biology Programme, Spanish National Cancer Research Centre (CNIO), Melchor Fernández Almagro, 3, E-28029 Madrid, Spain; 3 The Spanish Center of Rare Diseases (CIBERER U-731), Baldiri Reixac 10, E-08028 Barcelona, Spain; 4 Electronic and atomic protein modelling group, Barcelona Supercomputing Center, Plaça d’Eusebi Güell, 1-3, E-08034 Barcelona, Spain; 5 Clinical Biochemistry Department, Sant Joan de Déu Research Institute, Pg. de Sant Joan de Déu, 2, E-08950 Esplugues de Llobregat, Spain; 6 Nostrum Biodiscovery, Av. de Josep Tarradellas, 8-10, E-08029 Barcelona, Spain; 7 Physiological Sciences Department, Genetics Area, School of Medicine and Health Sciences, University of Barcelona, Bellvitge Campus. Feixa Llarga s/n, E-08907 L’Hospitalet de Llobregat, Spain; 8 Human Molecular Genetics Laboratory, Gene, Disease and Therapy Program, IDIBELL, Hospital Duran i Reynals, Avd. Gran Via de L’Hospitalet 199, E-08908 L’Hospitalet de Llobregat, Spain; 9 Department of Biochemistry and Molecular Biomedicine, University of Barcelona, Av. Diagonal, 643, E-08028 Barcelona, Spain

**Keywords:** Cryoelectron microscopy, Membrane proteins

## Abstract

Recent cryoEM studies elucidated details of the structural basis for the substrate selectivity and translocation of heteromeric amino acid transporters. However, Asc1/CD98hc is the only neutral heteromeric amino acid transporter that can function through facilitated diffusion, and the only one that efficiently transports glycine and D-serine, and thus has a regulatory role in the central nervous system. Here we use cryoEM, ligand-binding simulations, mutagenesis, transport assays, and molecular dynamics to define human Asc1/CD98hc determinants for substrate specificity and gain insights into the mechanisms that govern substrate translocation by exchange and facilitated diffusion. The cryoEM structure of Asc1/CD98hc is determined at 3.4–3.8 Å resolution, revealing an inward-facing semi-occluded conformation. We find that Ser 246 and Tyr 333 are essential for Asc1/CD98hc substrate selectivity and for the exchange and facilitated diffusion modes of transport. Taken together, these results reveal the structural bases for ligand binding and transport features specific to human Asc1.

## Abstract (web summary)

Asc1/CD98hc is a key regulator of small neutral amino acid transport in the brain and adipose tissue. Here, authors report the structure of semi-occluded hAsc1/CD98hc and provide a model for Asc1 exchange and facilitated diffusion modes of transport.

## Introduction

The amino acid transporter Asc1/CD98hc (SLC7A10/SLC3A2) is a non-stereoselective small neutral amino acid exchanger (e.g., glycine and, L- and D-serine, alanine, cysteine, and threonine)^1–4^ expressed mainly in the brain and adipose tissue^1,2,5^. Asc1/CD98hc belongs to the family of Heteromeric Amino acid Transporters (HATs), which are composed of a transporter (SLC7; L-amino acid transporter; LAT) and an ancillary (SLC3) subunit linked by a disulfide bridge^6^. Interestingly, Asc1/CD98hc is the only HAT for neutral amino acids that is not an obligatory exchanger, and it also mediates the release of intracellular substrates by facilitated diffusion^1,7,8^. In the exchange mode of transport, HATs switch between inward-open and outward-open conformations only concomitantly with the transport of a substrate. However, Asc1/CD98hc can transit from an outward-open to an inward-open conformation, or vice versa, without transporting an amino acid in the diffusion mode. The exchange mode of transport allows the concentration of pools of intracellular amino acids to be regulated by exchange with external amino acids^9^, whereas diffusion could bring about leakage, thereby uncoupling the concentrations of Asc1 substrates across the cell membrane.

Asc1/CD98hc is also the only member of the HAT family that efficiently transports glycine, and D- and L-enantiomers of the amino acid serine, and this particular substrate selectivity underlies the physiological functions of this transporter in both the central nervous system and adipose tissue^5,10^. Asc1/CD98hc mediates tonic D-serine and glycine release, which is required for optimal N-methyl D-aspartate receptor (NMDAR) activation in the hippocampus and cortical brain synapsis^3,8,11^. The role of Asc1/CD98hc in the central nervous system has prompted the search for inhibitors^3,12,13^ as potential drugs to treat the L-glutamate excitotoxicity present in stroke, amyotrophic lateral sclerosis, and trauma^14,15^. However, the lack of experimentally determined Asc1/CD98hc structures and thus of an understanding of the mechanisms of substrate selectivity have impeded drug development for clinical use. Asc1/CD98hc is also gaining momentum as a key metabolic regulator of adipose tissue with an impact on obesity and insulin resistance^5,16,17^. Glycine and L-serine uptake by Asc1/CD98hc to support glutathione levels and mitochondrial respiration is suspected to underlie the role of Asc1/CD98hc in adipocyte resilience to nutrient and oxidative stress. In addition, Asc1 has been identified as a white adipocyte marker that regulates D-serine intracellular content and controls preadipocyte differentiation^18^.

In the last few years, the structure of several HATs has been determined^19–28^, shedding light on the molecular mechanisms of substrate selectivity and translocation of this family of transporters^6,29^. However, an experimental structure of human Asc1/CD98hc at sufficient resolution to understand the mechanisms responsible for its unique functional features among HATs (lack of stereoselectivity and substrate diffusion) is not yet available.

Here, we report the structure of human Asc1/CD98hc determined by cryo-electron microscopy (cryoEM) at 3.4–3.8 Å resolution in a semi-occluded inward-facing conformation, which, together with substrate docking, molecular dynamics (MD) simulations, and functional studies, allows us to identify structural determinants for substrate selectivity and key residues involved in substrate translocation by exchange and facilitated diffusion. The structure provides information about the molecular basis of ligand binding and transport that are unique to Asc1/CD98hc, which would be helpful for structure-based drug design.

## Results

### CryoEM of human Asc1/CD98hc

**Fig. 1:**
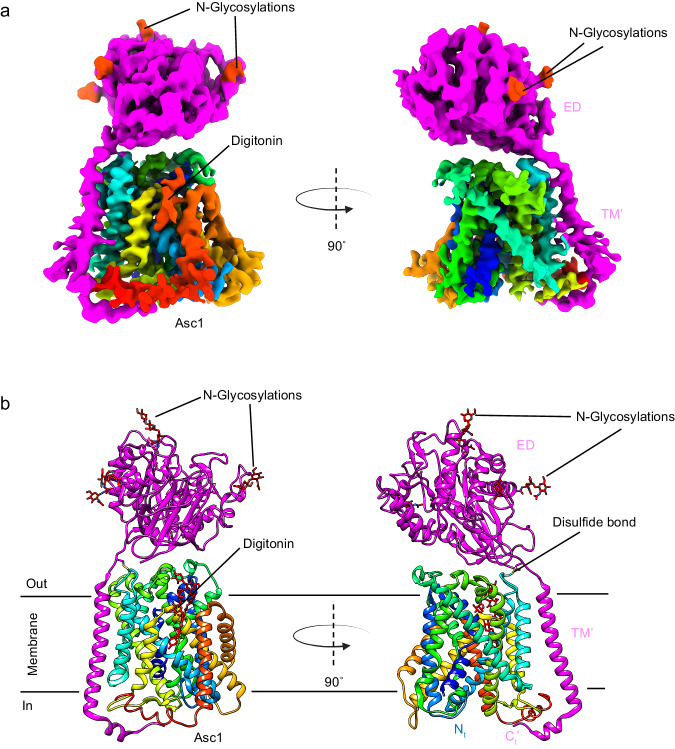
CryoEM structure of the Asc1/CD98hc heterodimer. **a** CryoEM map for Asc1/CD98hc heterodimer. Heavy chain CD98hc is colored in bright pink where the four N-glycosylations have been highlighted in red. Light chain is colored in rainbow from dark blue, blue, cyan, green, yellow, red, from N- to C-terminus. Densities assigned to glycosylations and the digitonin molecule are colored in red and labeled accordingly. The cryoEM map is shown in two different views, which differ approximately 90˚ from each other. **b** Atomic model of the Asc1/CD98hc heterodimer in the same orientation and color-code than in (**a**). The disulfide bond that covalently binds CD98hc (C109) to Asc1 (C154) is indicated.

We determined the structure of the human Asc1/CD98hc heterodimer in the absence of substrate in an inward-open conformation with a partial occlusion of TM1a (Fig. 1, Supplementary Table 1), as described in detail below. After expression in HEK293-6E cells, Asc1/CD98hc was purified by NiNTA affinity chromatography and a final step using size exclusion chromatography (Supplementary Fig. 1). The peak fraction of the size exclusion chromatography was applied to holey grids and vitrified, and cryoEM data were collected (Supplementary Fig. 2). Reference-free 2D averages of the extracted particles revealed that most of the ~140 kDa complex was made of well-defined transmembrane helices inserted within the detergent micelle, whereas an ectodomain was placed outside the micelle (Supplementary Fig. 2). We further classified the dataset by using iterative 3D reconstructions and classifications to yield a subset of particles that were refined to high resolution by removing the contribution of noise from the micelle using non-uniform refinement^30^. The estimated average resolution for the cryoEM map of Asc1/CD98hc was ~ 4 Å, with local resolutions as good as 3.4–3.8 Å for most of the map, except for some flexible cytoplasmic regions (Supplementary Fig. 2g), thereby allowing the modeling of the Asc1/CD98hc heterodimer structure (Supplementary Fig. 3, 4).

### Structure of inward-open semi-occluded Asc1/CD98hc

The cryoEM map for Asc1/CD98hc is in agreement with other reported HATs. It consists of a large density corresponding to the extracellular ectodomain of CD98hc sitting on top of Asc1, whilst Asc1 is mostly formed by transmembrane (TM) helices embedded in the detergent micelle (Fig. 1a). CD98hc contains a TM helix that expands across the membrane and interacts with the cytosolic side of Asc1 (Supplementary Fig. 4a, b). Additionally, the map shows density for the four N-glycosylation sites in CD98hc (Supplementary Fig. 4c). For Asc1, clear density was observed for all 12 TM helices of the transporter, with a lower resolution in TMs 1, 6, 11 and 12, thereby suggesting a certain degree of flexibility in these areas (Supplementary Fig. 2).

The structure of the heterodimer was confidently modeled for residues 41–489 for Asc1 and 61–529 for CD98hc (see Methods for details), thus excluding the flexible N-terminal ends of both proteins (Fig. 1b). Asc1 is covalently bound to CD98hc via a disulfide bridge involving residues Cys 154 (Asc1) and Cys 109 (CD98hc) (Fig. 1b, Supplementary Fig. 4a), with additional contacts between CD98hc TM’ helix and the surrounding TM helices of Asc1. The four N-glycosylation sites of CD98hc (residues 264, 280, 323, and 405) were also modeled (Supplementary Fig. 4c). Finally, density for a digitonin molecule was observed near TM helices 3, 9, 10, and 12 (Fig. 1), as previously reported for human LAT2^21^.

**Fig. 2:**
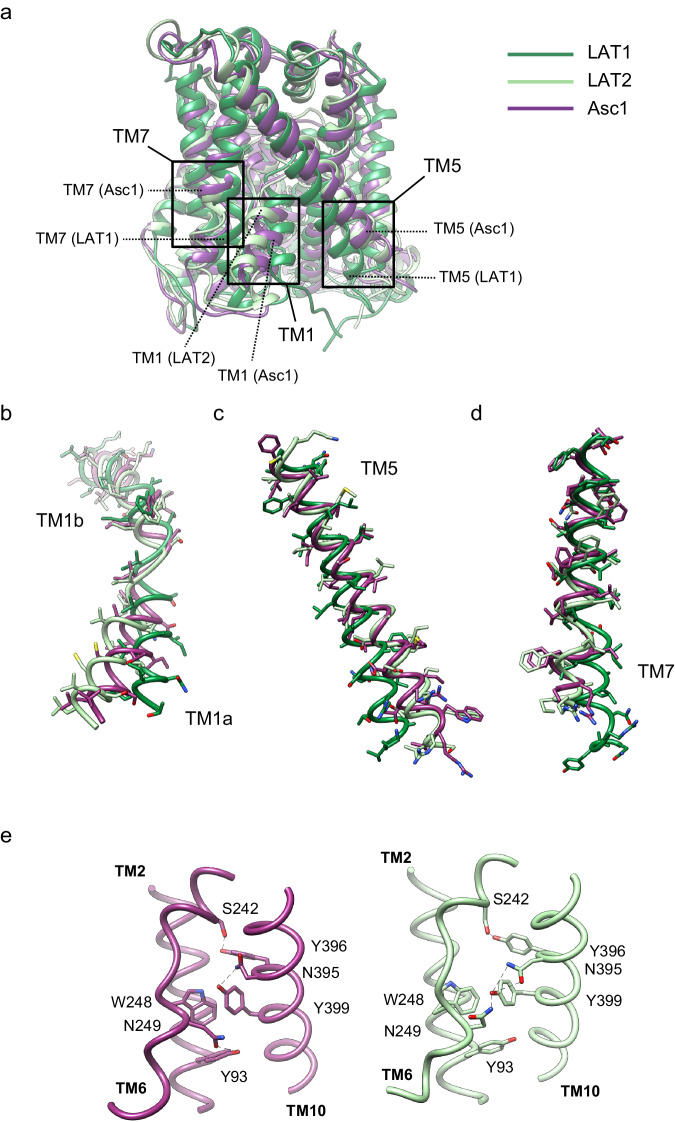
Inward-open partially occluded conformation of Asc1 and comparison with other LATs. **a** comparison between the structure of Asc1 (this work) with the structures of LAT1 (PDB ID 7DSQ) and LAT2 (PDB ID 7B00). Models are color-coded as follows: LAT1 in dark green, LAT2 in light green and Asc1 in dark purple. The regions of major differences are highlighted within squares and these regions are enlarged in (**b**−**d**). **b** Details of the differences found in TM1a and TM1b. **c** Details of the differences found in TM5. **d** Details of the differences in TM7. **e** Changes observed in TM10 as scaffold of TM6 between the structures of LAT2 and Asc1.

The structure of the Asc1/CD98hc heterodimer has been determined in an inward-open partially occluded conformation where TM1a is displaced towards TM5 when compared to the LAT2/CD98hc structure reported in a fully inward-open conformation (PDB ID 7B00)^21^ (global rmsd of backbone-only between Asc1 and LAT2 of 1.73 Å) (Fig. 2a, b). In contrast, Asc1/CD98hc is not as closed as shown for the fully occluded conformation of LAT1/CD98hc^25^ (PDB ID 7DSQ, global rsmd of 3.42 Å) where TM1a and TM5 block the access to the binding cavity from the cytosolic side (Fig. 2b, c). In addition, TM7 in Asc1 is in a position comparable to that of LAT2, thus differing from TM7 in the occluded structure of hLAT1 where TM7 is displaced towards TM1a (Fig. 2d). Therefore, the tilting of TM1a in Asc1 is the main conformational change responsible for partial occlusion of the access to the substrate cavity from the cytosolic side. In addition, we also found differences in a region that connects TM2, TM10, and TM6 in LAT2 and which has been proposed to contribute to maintaining the conformation of TM6^21^. Thus, Asn 249 in TM6b interacts with Tyr 399 in TM10 in the inward-facing conformation of LAT2, whereas Asn 249 is at putative H-bond distance of the side chain of Tyr 93 in TM2 in the partially occluded conformation of human Asc1 (Fig. 2e). This change in the connections established by Asn 249 likely results from the tilting of TM1a observed in Asc1 as the three residues involved are conserved in both LAT2 and Asc1.

We then compared the experimental structure of Asc1 with the AlphaFold structural model^31,32^ (Supplementary Fig. 5a). The prediction matched the overall structural organization of human Asc1 well (with a global rsmd of 1.67 Å over the 456 built residues). However, interestingly, discrepancies between the AlphaFold model and the experimental structure were found in regions involved in the function of the transporter: (i) TM1a is less occluded in the structure than in the prediction (Supplementary Fig. 5b) and the position of the backbone of Ile 53 in the C-terminal end of TM1a differs between the experimental structure and the prediction (Supplementary Fig. 5c); (ii) the side chain of Tyr 333 (TM8), a residue that is part of the canonical substrate binding site, is at potential H-bond distance of the side chain of Ser 246 (TM6) in the structure (see later) but the distance between these two residues is much larger in the model predicted by AlphaFold (Supplementary Fig. 5d); and (iii) Lys 194, a key functional lysine residue in TM5^33^ that is conserved in all LATs and the related cationic amino acid transporter GkApcT^34^, establishes a H-bond with backbone atoms of TM1 in all solved LAT structures in inward-facing conformation^19,21–23,28^ and also in the AlphaFold prediction for Asc1. However, the side chain of residue Lys 194 is not visible in the cryoEM map of Asc1 despite being surrounded by other well-defined residues in TM5 (Supplementary Fig. 5e), thereby suggesting that it does not establish contacts with TM1. This observation correlates well with the fact that the segment of TM1 facing Lys 194 was determined at a lower resolution than surrounding regions of the cryoEM map of Asc1, thus possibly indicating the flexibility of this region in the human Asc1 inward-facing partially occluded structure (Supplementary Fig. 5f).

### The Asc1 binding site contains a unique tyrosine in HATs

**Fig. 3:**
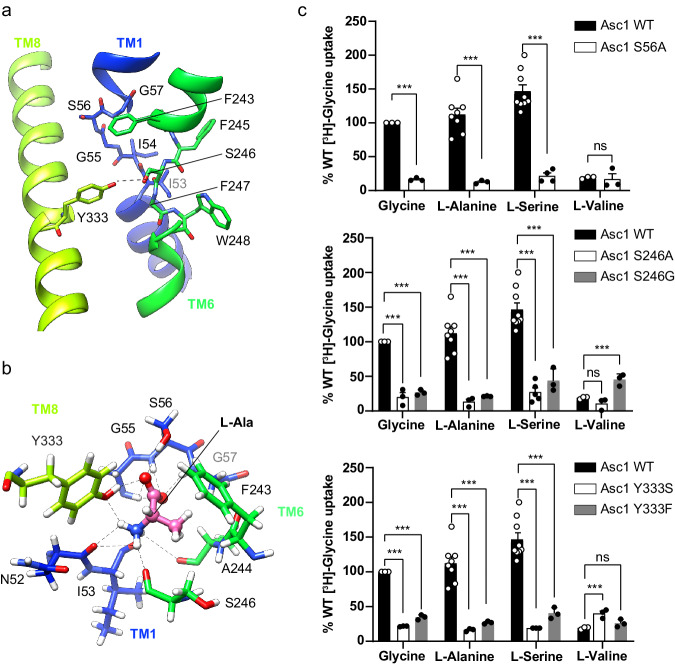
Asc1 substrate binding site and key residues for transport. **a** The unwound segments of TM1 and TM6 plus Phe 243 in TM6 and Tyr 333 in TM8 constitute the substrate binding site. The possible hydrogen bond between Ser 246 and Tyr 333 is indicated with a dashed line. **b** L-alanine pose 1 in the binding site with the lowest binding energy calculated by PELE. Putative H-bonds are indicated by dashed lines. C atoms in Asc1 residues are colored according with the color of the corresponding TMs as in Fig. 1, substrate C atoms are colored in pink. Another minimum for L-alanine (pose 2) is shown in Supplementary Fig. 8a. **c** Uptake of the indicated substrates in HeLa cells induced by wild-type (WT) and the indicated Asc1 mutants. Uptake activity is normalized for glycine uptake mediated by WT Asc1/CD98hc (2073 ± 223 pmol mg^−1^ protein min^−1^ from 17 independent experiments). Data (mean ± s.e.m.) corresponds to *n* = 9 (WT L-Ser), *n* = 8 (WT L-Ala), *n* = 5 (S246A L-Ser), *n* = 4 (S56A L-Ser) or *n* = 3 (rest of conditions) independent experiments. *P* values were obtained by fitting a linear model to Boxcox transformed data and were adjusted for multiple comparisons using the Benjamini-Hochberg method (see materials and methods): ns, non-significant; (***), *p* < 0.001. WT vs S56A Gly, *p* = 7.11 ∙ 10^−15^; WT vs S56A L-Ala, *p* = 8.88 ∙ 10^−16^; WT vs S56A L-Ser, *p* < 2.22 ∙ 10^−16^; WT vs S56A L-Val, *p* = 0,0492; WT vs S246A Gly, *p* = 2.34 ∙ 10^−10^; WT vs S246A L-Ala, *p* = 1.37 ∙ 10^−12^; WT vs S246A L-Ser, *p* < 2.22 ∙ 10^−16^; WT vs S246A L-Val, *p* = 0,0994; WT vs S246G Gly, *p* = 1.11 ∙ 10^−14^; WT vs S246G L-Ala, *p* = 3.42 ∙ 10^−14^; WT vs S246G L-Ser, *p* = 1.15 ∙ 10^−10^; WT vs S246G L-Val, *p* = 1.89^10-5^; WT vs Y333S Gly, *p* = 4,44 ∙ 10^−16^; WT vs Y333S L-Ala, *p* = 6.66 ∙ 10^−16^; WT vs Y333S L-Ser, *p* = 3.11 ∙ 10^−15^; WT vs Y333S L-Val, *p* = 3.82 ∙ 10^−4^; WT vs Y333F Gly, *p* = 9.53 ∙ 10^−12^; WT vs Y333F L-Ala, *p* = 1.92 ∙ 10^−12^; WT vs Y333F L-Ser, *p* < 1.65 ∙ 10^−10^; WT vs Y333F L-Val, *p* = 0.153.

The substrate binding site of the human Asc1 transporter is in an inward-facing partially occluded conformation, with TM1a tilted to limit the access of substrates to the binding site from the cytosolic side of the membrane. As previously reported for other LATs, the substrate coordination site is formed by the unwound regions of TMs 1 and 6^19,21–23,28^, together with other residues in TMs 1a, 6b and 8 (Fig. 3a). In particular, TM1 contains a ^55^G(S/T)G^57^ motif with the amide nitrogen atoms of Gly 55, Ser 56 and Gly 57 oriented towards the empty space, providing the possibility of hydrogen bonding with the carboxyl group of substrates. In addition, the unwound segment of TM6 includes Ser 246 with its carbonyl group facing the binding cavity, and Trp 248 facing residues in the adjacent TM2 and TM10 (Fig. 3a). Together, these residues in the unwound regions of TM1 and TM6 generate a cavity with the correct environment for the coordination of the carboxyl and amine groups of substrates.

In Asc1, the substrate binding site is also complemented by Tyr 333 located in TM8 (Fig. 3a), a residue only present in this transporter within the HATs and found to be important for the mechanism of substrate selectivity and translocation (see below). The hydroxyl groups of Tyr 333 and Ser 246 are at potential H-bond distance, and they delimit one of the edges of the substrate binding cavity (Fig. 3a, Supplementary Fig. 5d).

Interestingly, Asc1 appeared later in evolution than other metazoan LATs, being found only in vertebrates and coinciding with the appearance of Tyr 333 in the substrate binding site of Asc1. Tyr 333 is fully conserved in all available Asc1 sequences (Supplementary Fig. 6). This Tyr residue is also present in BasC, a bacterial LAT transporter with Asc-like transport activity^33^. This observation thus suggests that this residue contributes to functions in substrate binding and/or transport that are specific to Asc1 among vertebrate LATs, such as the transport of D-serine.

### TM1, TM6 and TM8 contribute to Asc1 substrate recognition

Despite the addition of 10 mM D-serine to the purified heterodimer before sample vitrification, we found no density for the ligand in the putative binding site. To overcome this limitation, we performed molecular docking and PELE studies^35^ using the structure of Asc1/CD98hc and a set of amino acids that are substrates for Asc1 (L-alanine, L-serine, and D-serine). The PELE analysis starts by docking the substrate inside the binding cavity, which then can explore different binding poses, thus overcoming the lack of bound substrate in our structure. This approach predicted minimal binding-energy modes for all the amino acids tested (Supplementary Fig. 7). In the calculated poses, the α-carboxyl of these substrates are predicted to establish H-bonds with the nitrogen atom of residues Gly 55, Ser 56, and Gly 57 in the GSG motif of the unwound segment of TM1, and with the hydroxyl group of Ser 56 in TM1. In contrast, the α-amino of the substrates are predicted to establish H-bonds with carbonyl oxygen atoms of Asn 52 and/or Ile 53 in TM1, as well as with Ser 246 in TM6 (Fig. 3b, Supplementary Fig. 8a–d). Furthermore, each substrate established additional interactions with specific residues in TM6 and TM8, as discussed below.

PELE analysis revealed two minima for L-alanine (Supplementary Fig. 7). In the lowest-energy pose (pose 1), the side chain of Tyr 333 interacted with both the α-carboxyl and α-amino groups of the substrate (Fig. 3b). In the other PELE pose (pose 2), the side chain of Tyr 333 was oriented towards TM6, causing a shift in the interaction network of the substrate, which is then predicted to establish H-bonds with the side chain of Ser 246 (Supplementary Fig. 8a).

In the case of L-serine and D-serine, PELE analysis revealed that the α-amino and α-carboxyl groups of both substrates share similar interaction networks (Supplementary Fig. 8b–d). Nevertheless, some differences were observed in the side-chain hydroxyl interactions. For L-serine, we obtained two poses with similar binding energy values (Supplementary Fig. 7): one where the substrate hydroxyl group interacted with Phe 243 in TM6 (Supplementary Fig. 8c) and another where it interacted with Tyr 333 in TM8 (Supplementary Fig. 8d). D-serine presented a single minimum where the substrate hydroxyl group interacted with Phe 243 (Supplementary Fig. 8b).

Our PELE studies indicated that human Asc1 binds its substrates between the unwound regions of TM1 and TM6 and identified a series of residues in TM1 (Ser 56), TM6 (Ser 246) and TM8 (Tyr 333) whose side chains might participate in substrate recognition. Remarkably, in Asc1 a putative interaction has been observed between the substrate and TM8, thus connecting the bundle (TMs 1, 2, 6, and 7) and scaffold (TMs 3, 4, 8, and 9) domains, a similar design to that previously reported for the binding of L-arginine in the prokaryotic homolog AdiC^36^.

### Ser 56, Ser 246 and Tyr 333 are determinants of selectivity

PELE analysis identified several residues whose side chain interacts with the substrate in the binding site (Ser 56, Ser 246, and Tyr 333). Thus, we performed functional studies of Asc1 mutants predicted to interfere with substrate binding (S56A, S246A, S246G, Y333S, and Y333F). To this end, we measured amino acid uptake in HeLa cells co-transfected with either the human wild-type or the mutated version of Asc1, together with human CD98hc. The expression levels and subcellular localization of these Asc1 mutants were comparable to those of the wild-type transporter (Supplementary Fig. 9). All these mutations caused a dramatic decrease in the uptake of small neutral substrates ([^3^H] glycine, [^3^H] L-alanine and [^3^H] L-serine) but did not affect (in the case of S56A and S246A) or slightly increased (in the case of Y333F, Y333S and S246G) the uptake of the larger amino acid ([^3^H] L-valine) (Fig. 3c). These results indicate that Ser 56, Ser 246 and Tyr 333 establish interactions with the substrates that are essential for the transport of small amino acids but not for larger ligands (e.g., branched-chain amino acids) such as L-valine, for which Asc1/CD98hc is a much poorer transporter^1^. L-valine might require the participation of other residues in Asc1.

Asc1 shows distinctive properties in its acceptance of D-amino acids^1^, although the molecular bases underlying this lack of stereoselectivity have not been yet described. Consequently, the transport of [^3^H] L- and D-serine in the S56A, S246A, S246G Y333S, and Y333F mutants of Asc1 co-expressed with CD98hc was studied in HeLa cells. We observed a comparable decrease in the uptake of both enantiomers, in agreement with the highly similar interaction networks that these two substrates shared in the PELE binding poses (Supplementary Fig. 8e).

Taken together, our results suggest that the residues studied (Ser 56, Ser 246, and Tyr 333) are essential for the transport of small neutral amino acids and that point mutations of these residues do not alter the selectivity of the transporter towards the enantiomers of serine. We hypothesize that evolution has sculpted the binding site of Asc1 to transport small neutral amino acids in a non-stereoselective manner, with three essential interactors that are shared between serine enantiomers. Having established that these residues are essential for transport activity, we then proceeded to investigate further into their possible mechanistic role in the two transport modes presented by Asc1, namely facilitated diffusion and exchange.

### Tyr 333 is important for facilitated diffusion

Asc1/CD98hc is the only HAT of neutral amino acids that presents two distinct transport modes, namely amino acid exchange and facilitated diffusion^1,7,8^. Considering that Tyr 333 is a residue found only in Asc1 within vertebrate LATs (Supplementary Fig. 6), that the hydroxyl groups of Tyr 333 and Ser 246 are predicted to be at possible H-bond distance in our structure (Fig. 3a, Supplementary Fig. 5d), and that this potential bond would connect the scaffold and bundle domains in an analogous manner to the substrate in our PELE analysis (Fig. 3b, Supplementary Fig. 8a–d), we hypothesized that these residues participate in the facilitated diffusion mode of transport of Asc1. MD analysis of our apo structure predicted that the interaction between the side chains Ser 246 and Tyr 333 is weak, being present in less than 2% of the frames of the simulation (Supplementary Fig. 10). Nevertheless, given that our structure in a partially occluded conformation is likely to represent a transitory state between more stable states and that we cannot rule out a stronger interaction between Tyr 333 and Ser 246 in another conformation (i.e., fully occluded), we performed functional assays to obtain further insight into the possible role of this connection in facilitated diffusion.

To this end, we set up a system to measure and compare amino acid transport by exchange and by facilitated diffusion in HeLa cells overexpressing wild-type Asc1/CD98hc and selected mutants (S246G and Y333F) (Supplementary Fig. 11). The efflux of radioactivity from cells preloaded with [^3^H] D-serine was measured in linear conditions in the following three conditions (Supplementary Fig. 11a–d): (i) in the presence of the specific Asc1 inhibitor BMS466442 at full-blocking concentration of 5 µM^12^ to measure efflux of radioactivity not mediated by Asc1/CD98hc (Supplementary Fig. 11b). We consistently detected less than 20% of the loaded radioactivity coming out from inside the cells in the presence of the inhibitor, which was considered as experimental background (Supplementary Fig. 11e, f); (ii) in the absence of amino acid outside the cells (Supplementary Fig. 11c), radioactivity could only be transported by Asc1 from the cytosol to the extracellular space by facilitated diffusion, and these conditions were achieved by performing the experiments in an amino acid-free medium (Supplementary Fig. 11f); (iii) in the presence of extracellular D-serine at saturating concentrations (3 mM; 15–20-fold K_m_ value) (Supplementary Fig. 11d), to measure transport by the exchange mechanism of Asc1 (Supplementary Fig. 11e). In these two latter cases, to estimate the transport by Asc1 corresponding to facilitated diffusion or exchange, we subtracted the background measured in the presence of the Asc1/CD98hc inhibitor performed in parallel from each measurement.

**Fig. 4:**
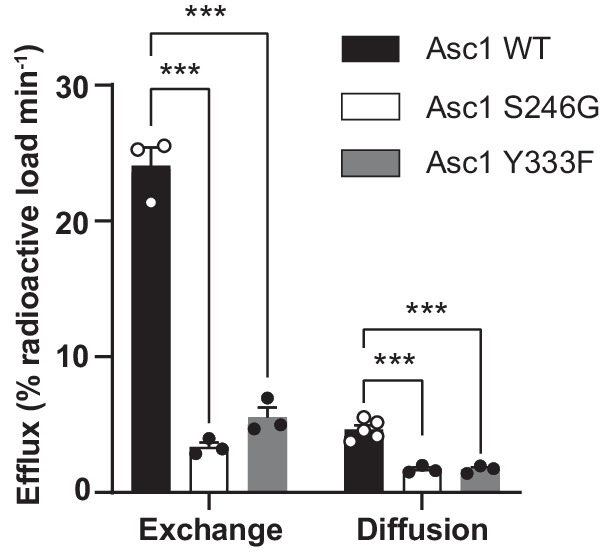
Ser 246 and Tyr 333 play a key role in the transport function of Asc1. Efflux quantification in percentage of the loaded radioactivity per minute in HeLa cells transfected with wild-type Asc1 or mutants S246G and Y333F. Data (mean ± s.e.m.) corresponds to *n* = 5 (WT facilitated diffusion) or *n* = 3 (rest of efflux measurements) independent experiments. A linear model was fitted to transformed data (see material and methods) to obtain *p* values comparing wild-type Asc1 with each mutant. *P* values were not adjusted for multiple comparisons. (***) *p* < 0.001. WT vs S246G exchange, *p* = 5.76 ∙ 10^−10^; WT vs S246G diffusion, *p* = 8.30 ∙ 10^−7^; WT vs Y333F exchange, *p* = 2.45 ∙ 10^−8^; WT vs Y333F diffusion, *p* = 7.72 ∙ 10^−7^.

Efflux via Asc1 was expressed as the percentage of the loaded radioactivity released to the medium per minute after subtraction of the background measured in the presence of the inhibitor (Fig. 4). In all the experimental conditions (wild-type Asc1, and S246G and Y333F mutants), the intracellular content of Asc1 substrates as a whole in HeLa cells was not altered after loading with [^3^H] D-serine (Supplementary Fig. 11h). Efflux via wild-type Asc1 by diffusion was ~19% of that calculated by exchange (Fig. 4). Interestingly, facilitated diffusion in the S246G and Y333F mutants fell to 36%, and exchange to 14% and 23% of the wild-type values, respectively (Fig. 4).

The first step of the efflux experiments consisted of binding the radiolabeled substrate to the Asc1 transporter in an inward-facing conformation. A conformational transition to outward-facing conformation then took place and substrate was released to the extracellular space. Finally, in the exchange mode, the return to the inward-facing conformation occurred with the external substrate (D-serine) bound to Asc1, whereas this happened in the absence of substrate in the diffusion mode (Supplementary Fig. 11a). By using the mathematical model described in *Methods*, we estimated the difference between the time the transporter takes to return from the outward- to the inward-facing conformation, either empty (diffusion) or bound to D-serine (exchange) to complete the efflux of 1% of the loaded radioactivity (t_IN_D-t_IN_E), for the wild-type Asc1 and mutants (Table 1, Supplementary Fig. 11g). This time difference was 1.5 and 2.6-fold higher in the S246G and Y333F mutants, respectively, when compared to wild-type Asc1 (Table 1). This observation could be explained by a slower outward to inward transition of the empty transporter (which corresponds to an increase in t_IN_D values) or to a faster outward to inward transition of the substrate-bound transporter (corresponding to a decrease in t_IN_E values), or both. The most likely scenario is a reduction in the velocity of the return of the empty transporter from outward- to inward-facing conformations because mutants S246G and Y333F dramatically decreased efflux by exchange (Fig. 4) and the V_max_ of D-serine uptake in HeLa cells (Supplementary Fig. 8f).

**Table 1:** Time difference between the inward return of the transporter in the diffusion and exchange modes of transport of wild-type Asc1 and the indicated mutants

	t_IN_^D^ - t_IN_^E^ (min per % of radioactive load)
hAsc1 WT	0.182 ± 0.018
hAsc1 S246G	0.284 ± 0.072 (*)
hAsc1 Y333F	0.475 ± 0.084 (**)

Time for efflux by diffusion minus Time for efflux by exchange (t_IN_D – t_IN_E) (min per 1% of loaded radioactivity^−1^), which corresponds to the time to accomplish the return of the transporter to the inward-facing conformation in the diffusion mode (t_IN_D) – minus this time in the exchange mode (t_IN_E). Data (mean difference ± s.e.m.d.) corresponds to *n* = 5 (WT facilitated diffusion) or *n* = 3 (rest of efflux measurements) independent experiments of HeLa cells transfected with wild-type Asc1 or mutants S246G and Y333F. A linear model was fitted to transformed data (see material and methods) to obtain *p* values comparing wild-type Asc1 with each mutant, and between the two mutants. *P* values were not adjusted for multiple comparisons. (*) *p* < 0.05; (**) *p* < 0.005. WT vs S246G t_IN_D – t_IN_E, *p* = 0.0486; WT vs Y333F t_IN_D – t_IN_E, *p* = 0.00165.

**Fig. 5:**
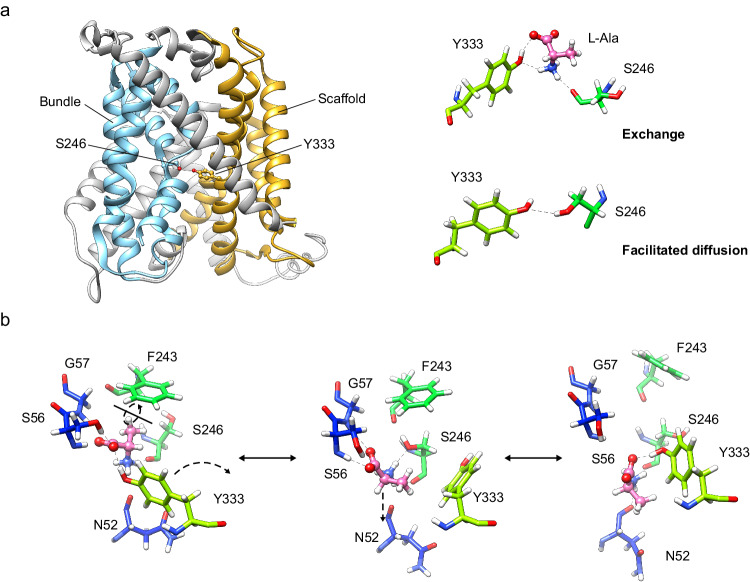
Proposed model for the mechanistic roles of Ser 246 and Tyr 333 in Asc1 transport. **a** In the exchange mode of transport, the substrate (L-alanine) intercalates between Ser 246 and Tyr 333, thus bridging the scaffold (tinted in orange) with the bundle domain (tinted in cyan) of the transporter. Analogously, in the facilitated diffusion mode of transport, Ser 246 and Tyr 333 form a potential hydrogen bond between the side chains. Our results support a mechanism in which the connection between Ser 246 and Tyr 333 triggers the fully occluded conformation in the exchange mode of transport, with the transporter bound to substrate, but also in the diffusion mode, where the transporter cycles back with an empty binding cavity. **b** Proposed role of Ser 246 and Tyr 333 in facilitating the transition of the substrate to and from the transport-productive binding pose. In this model, the productive pose of the substrate between TM1 and TM6 (left, corresponding to the PELE pose 1 shown in Fig. 3b) would be destabilized by a rotamer of Tyr 333 towards TM6 (right, corresponding to the second MD replicate at 24 ns), which would have the effect of pulling the substrate away from the GSG motif in TM1, a necessary step for the release of the substrate to the cytosol. The side chain of Ser 246 would stabilize the intermediate step (middle, corresponding to the PELE pose 2 shown in Supplementary Fig. 8a), in which Tyr 333 has rotated but not yet re-bound to the substrate and the substrate itself has experimented a ~ 180° rotation. C atoms in Asc1 residues are colored according with the color of the corresponding TMs as in Fig. 1 whereas substrate C atoms are coloured in pink.

In all, our results suggest that Ser 246 and Tyr 333 are relevant both for facilitated diffusion and exchange. PELE analysis predicted the intercalation of substrates between these two residues in the transporter, thus bridging the bundle (TM1 and TM6) and scaffold domains (TM8). Tyr 333 might help coordinate these two domains during the occlusion of the transporter (Fig. 5a). Tyr 333 is unique to Asc1 within the light subunits of HATs, and this observation reinforces the notion that this residue contributes to facilitated diffusion as a mode of transport for HATs of neutral amino acids.

### Mechanistic insights into substrate translocation in Asc1

Both our uptake and efflux assays of the Asc1 binding site mutants support the notion that Ser 56, Ser 246, and Tyr 333 are essential for the exchange mode of transport. To further explore the mechanistic role of these residues in amino acid exchange, we performed MD analysis with the L-alanine-bound semi-occluded transporter using the lowest-energy PELE binding pose as the starting point (Fig. 3b).

In two of the three replicates, the substrate remained solidly bound between TM1 (backbones of Ile 53, Ser 56 and Gly 57, as well as the side chain of Ser 56) and TM6 (backbone of Ser 246), whereas Tyr 333 rapidly dissociated from the substrate and showed only a weak interaction (<7% of the frames of the simulation) (Supplementary Fig. 12a). However, in a third replicate, Ser 56 and Tyr 333 competed for the interaction with the substrate α-carboxyl during an initial phase of the simulation (up to ~11 ns) (Supplementary Figs. 12b and 13a). This was followed by a rotation of the side chain of Tyr 333 towards TM6 (reminiscent of our PELE pose 2 shown in Supplementary Fig. 8a) that dragged the substrate away from Ser 56 and Gly 57 (Supplementary Figs. 12b and 13b). Strikingly, in the MD simulation, upon the disconnection of the substrate from the unwound region of TM1, the TM1a helix opened further the cytosolic vestibule (Supplementary Fig. 14a, b) and the substrate started to transition out of the binding site (Supplementary Fig. 14c). Once the substrate was definitively dissociated from Ser 56, it remained bound to Tyr 333, as well as to the backbones of Asn 52, Ile 53 (TM1a) and Ser 246 (TM6) (Supplementary Fig. 13b). The interaction with Tyr 333 was lost at 27 ns, concomitant with the binding of the substrate α-carboxyl with the ε-amine of Lys 194 (TM5) (Supplementary Fig. 13c). At 30 ns, the substrate lost interaction first with Ser 246 and immediately after with Asn 52 and Lys 194 (Supplementary Fig. 13d), at which point L-alanine definitively was released from Asc1.

These results are evocative of previous MD studies of the bacterial LAT BasC^33^, in which a transition of the substrate towards the equivalent TM5 residue to Asc1 Lys 194 precedes the release of the substrate from the transporter to the cytosol. Functional studies in BasC showed that mutation of this TM5 lysine residue to alanine has a severe impact on the external K_m_ and maximal velocity in BasC and Asc1^33^. Considering the interplay between Ser 56, Ser 246, and Tyr 333 in shuttling the substrate outside the binding site in our MD with human Asc1, we then proceeded to perform a kinetic analysis of [^3^H] L-alanine uptake to assess whether the mutation of these residues also impacts substrate recognition and translocation (Table 2, Supplementary Fig. 15). Indeed, L-alanine uptake by Asc1 S56A mutant showed an increased extracellular K_m_ and a decreased V_max_ compared to wild-type Asc1, thereby suggesting that this mutation impaired the binding energy and the translocation for L-alanine, thus affecting one or several transport-limiting steps. In contrast, the S246G (TM6) and Y333F (TM8) mutants substantially reduced V_max_ without affecting K_m_. This observation thus highlights the role of these two residues in substrate translocation. We further characterized the impact of mutating Ser 246 and Tyr 333, a pair of residues unique in Asc1 among HATs, on the kinetic parameters of transport of D-serine, a substrate uniquely transported by Asc1 among HATs. Much like for L-alanine, S246G and Y333F showed a dramatic reduction in the V_max_ of D-serine uptake. Moreover, the S246G mutant also showed a small increment in the K_m_ for D-serine (Supplementary Figs. 8f and 15). This observation suggests that the interaction of the hydroxyl group of Ser 246 with D-serine observed in our PELE analysis contributes to the binding energy of this substrate (Supplementary Fig. 8b).

**Table 2:** Kinetics parameters of L-alanine uptake induced by Asc1/CD98hc in HeLa cells

	L-alanine uptake	
	K_m_ (μM)	V_max_ (pmol mg^−1^ protein min^−1^)
hAsc1 WT	35 ± 11	4659 ± 370
hAsc1 S56A	88 ± 8 (***)	1105 ± 79 (***)
hAsc1 S246G	48 ± 6 (ns)	1590 ± 152 (***)
hAsc1 Y333F	33 ± 3 (ns)	1328 ± 178 (***)

Data (mean ± s.e.m.) of wild-type and the indicated mutants of Asc1 corresponds to *n* = 3 independent experiments run in triplicates. A linear model was used to compare conditions after log-transforming the data to obtain *p* values. *P* values were adjusted for multiple comparisons using the Benjamini-Hochberg method (see material and methods): ns, non-significant; (***) *p* < 0.001. WT vs S56A K_m_, *p* = 7.20 ∙ 10^−5^; WT vs S56A V_max_, *p* = 2.46 ∙ 10^−8^; WT vs S246G K_m_, *p* = 0.104; WT vs S246G V_max_, *p* = 5.93 ∙ 10^−7^; WT vs Y333F K_m_, *p* = 0.878; WT vs Y333F V_max_, *p* = 1.06 ∙ 10^−7^.

Taken together, our MD and kinetic analysis point towards a model where Ser 56 would fix the substrate in a transport-productive pose between the unwound segments of TM1 and TM6 (Fig. 5b), thus explaining why the mutation of this residue affects both K_m_ and V_max_, whereas the rotation of Tyr 333 towards TM6 would help to pull the substrate away from Ser 56 in order to transit in and out of this pose. This latter conformation was predicted by the PELE analysis for L-alanine, which also shows that the side chain of Ser 246 interacts with the substrate, perhaps also contributing to the pulling of the substrate towards TM6. Mutations of both Tyr 333 and Ser 246 have a drastic impact on uptake V_max_, thus supporting a mechanistic role of these residues on substrate translocation.

## Discussion

The physiological roles of Asc1/CD98hc in the central nervous system and adipose tissue make this particular HAT a potential target to treat brain disorders and obesity^1–3,5^. However, the Asc1/CD98hc structure and molecular bases that underlie its function remain unknown. Here we reveal the structure of Asc1/CD98hc in a partially occluded inward-facing conformation not previously described for any HAT and identify Tyr 333 and Ser 246 as key residues for substrate transport mechanisms in human Asc1/CD98hc.

The semi-occlusion of Asc1 is the result of a partial tilting of TM1a to an intermediate position between the fully open cytosolic gate of human LAT2^21^ and the fully occluded cytosolic gate of human LAT1^25^. As a result, the polar contact between the fully conserved lysine residue in the TM5 of LATs (Lys 194; human Asc1 numbering) with a backbone atom in TM1, present in all LAT structures already determined^19–24,26–28,33^, is not present in the semi-occluded inward-facing conformation of Asc1. Upon complete closing of the cytosolic gate, this conserved lysine residue bridges backbone atoms in TM5 and TM8, as shown in human LAT1^25^ and in the Cationic Amino Acid transporter homolog GkApcT^34^, but not in Asc1. The lack of density for the side chain of Lys194 in the cryoEM map of the semi-occluded Asc1 (Supplementary Fig. 5e), together with the low resolution observed for TM1, indicative of flexibility (Supplementary Fig. 5f), suggests that we captured a transient state of human Asc1 with partial tilting of TM1a towards the occlusion of the cytosolic gate of the transporter but without a defined position for the side chain of Lys194. Therefore, the semi-occluded state of human Asc1 may reflect a certain propensity to close the cytosolic gate in the absence of bound substrate—a characteristic not shared by LAT1^19,20,25^, LAT2^21^, xCT^22^, or b^0,+^AT^24^. Thus, this TM1a occlusion, which may hinder substrate accessibility, together with the intrinsic low affinity of inward-facing LATs and HATs for substrate^9,33,37^, may explain the absence of substrate in the solved structure, in spite of incubation with 10 mM D-serine prior to vitrification.

In addition, the semi-occlusion of TM1a results in a reorganization of the H-bond network responsible for the stabilization of the unwound region of TM6, which has been recently shown to play a role in the substrate selectivity profile of human LAT2 transporter^21^. In the semi-occluded conformation of Asc1, an interaction between TM2 and TM6 is mediated by Tyr 93 and Asn 249, thus stabilizing the unwound region of TM6 (Fig. 2e). MD simulations of Asc1 bound to L-alanine substrate suggested that the position of Asn 249 in TM6 would instead be stabilized by Tyr 399 in TM10 as a result of TM1a opening (Supplementary Fig. 14d), and this conformation would be similar to that observed in the inward-open structure of human LAT2^21^ (Fig. 2e).

The substrate binding site in all HATs is formed by unwound segments in TM1 and TM6, whose residues expose their amide and carbonyl backbone atoms, which serve as engaging platforms for the α-amino and carboxyl moiety of each substrate^19,23,28^. The core of this mechanism is conserved in human Asc1, including the ^55^G(S/T)G^57^ motif recognizing the carboxyl group of substrates. However, uniquely within HATs, the pair formed by Tyr 333 in TM8 and Ser 246 at the unwound segment of TM6 contributes to the particular substrate selectivity profile of Asc1, transporting small neutral D- and L- isomers, and being the only HAT able to transport glycine efficiently^1,2,21^. On the one hand, the interaction of the substrate with TM8 through Tyr 333 is unique among HATs, although other APC-fold transporters such as AdiC^36^ rely on substrate interaction with TM8 for transport function. In addition, the equivalent position of Tyr 333 in the prokaryotic SLC7 transporters BasC (Tyr 290)^33^ and GkApcT (Met 321)^34^ reduces the volume of the substrate binding site cavity, allowing the efficient uptake of small neutral amino acids but not larger ones. On the other hand, according to the predictions, Ser 246, a position previously reported to be involved in affecting selectivity for substrate size^20,21^, could also contribute to the interaction with substrates in the case Asc1 (Supplementary Fig. 8a, b).

The molecular mechanism underlying the role of Tyr 333 and Ser 246 in the transport activity of Asc1 is still unknown, but our results allow speculation. The possible H-bond between Ser 246 (TM6) and Tyr 333 (TM8) in the semi-occluded apo state of Asc1 and the insertion of the substrates between the two residues would serve to connect the bundle (TMs 1, 2, 6, and 7) and the scaffold (TMs 3, 4, 8 and 9) domains of Asc1. This connection might contribute to the closing of the cytosolic gate of the transporter in the absence of bound substrate, a step in the diffusion mode of transport, and with the substrate bound in the exchange mode of transport (Fig. 5a). In agreement with our results, docking and MD analysis also inserted the substrate D-serine between Ser 246 and Tyr 333 in an outward-facing occluded conformation model of Asc1 obtained by homology modeling from the structure of a distant bacterial APC transporter (AdiC)^29^. This observation supports the notion that the insertion of the substrate between Ser 246 and Tyr 333 goes along with the closing of the cytosolic gate. Interestingly, a similar connection between the bundle and the scaffold domains is key in the closing of the cytosolic gate of the APC-fold Neurotransmitter Sodium Symporter homolog LeuT^38^. Nevertheless, our MD analysis showed that the interaction between Ser 246 and Tyr 333 is not stable in the apo semi-occluded conformation of Asc1 (Supplementary Fig. 10). Whether this connection is stronger in other conformations of Asc1 and is, therefore, a contributing factor to inducing the transitions of the cytosolic and external gates without substrate binding, or whether different mechanisms underlie the role of these two residues in the diffusion mode of transport is still unknown.

Computational analysis of the Asc1 structure docked with L-alanine has shed light on other possible roles of Ser 246 and Tyr 333 in the transition of the substrate through the binding site (Fig. 5b). In this model, Tyr 333 would contribute to substrate release from the binding site through competition for the substrate with the ^55^GSG^57^ motif in TM1. Rotation of Tyr 333 towards TM6 would have a pulling effect on the substrate, which, upon disconnection from TM1, would then be primed for transfer towards Lys 194 and subsequently dissociate from the transporter. The side chain of Ser 246 could also contribute to disconnect the substrate from TM1, as suggested by the pose 2 for L-alanine in the PELE analysis (Supplementary Fig. 8a). Conversely, the same process in reverse could be proposed as the sequence of events happening during the transit of the substrate from the cytosol towards the transport-productive binding pose. In agreement with this model, the S246G and Y333F mutants, which would be unable to mediate substrate entry to or exit from the binding site, result in a reduction of V_max_ with minimal impact on K_m_.

All these observations point towards a model where Ser 246 and Tyr 333 trigger the occlusion of the cytosolic gate either by connecting the bundle and scaffold domains and/or by positioning the substrate in a productive pose within the substrate-binding site of Asc1. Determining structures of Asc1 in fully occluded or outward-facing conformations would shed light on the role of Tyr 333 and Ser 246 in the opening/closing of the transporter external gate. In this line, the recently solved structure of human LAT1/CD98hc in an outward-open and occluded conformations in lipid nanodiscs^39^, suggests the need to test various lipidic environments for exploring conformational transitions in Asc1/CD98hc.

## Methods

### Construct design and protein expression for cryoEM purposes

The heterodimer components were cloned in two independent mammalian pcDNA3.1(+) vectors and co-expressed in HEK293-6E mammalian cells (CVCL_HF20, licensed through the National Research Council of Canada) grown in suspension (37 °C, 5% CO_2_ atmosphere and humidity-controlled) using F17 Freestyle medium (Invitrogen, Life Technologies, Darmstadt, Germany) supplemented with 1 g L^−1^ pluronic F68 (Applichem, Darmstadt, Germany), 4 mM L-glutamine and 12.5 mg L^−1^ G418. The human light subunit Asc1 carried a 6xHis-tag at C-terminus, whereas CD98hc had a OneStrep-tag at N-terminus. For preliminary protein expression and purification trials, a C-terminal in-frame eGFP fused version of hAsc1 was used. Once the heterodimer expression and purification were reproducible and in order to reduce purification time, the GFP reporter protein was removed from the final construction. Cells were grown to 1.5 × 10^6^ cells ml^−1^ in 2 L Erlenmeyer flasks with ventilation membrane caps (Triforest Plasticware Irvine, CA, USA) with a working volume of 600 ml culture per flask. For transfection, PEI:DNA polyplexes were prepared by mixing a total of 1 μg of DNA (1:1 w/w hAsc1 and CD98hc) and 4 μg PEI-MAX 40000 (Polysciences Europe GmbH) per ml culture, diluting the mixture in 1 to10 of the total culture volume of fresh media. Polyplexes were allowed to form for 3 min at room temperature with intermittent mixing before addition to the cells. Finally, cells were collected 48 h after transfection, and the cell pellets were washed twice with 50 ml of PBS and then stored at −80 °C until use.

### Protein purification and grid preparation

Typically, the pellet collected from a 500 ml expression was sufficient for grid preparation. The heterodimer purification and grid preparation were carried out on the same day. Cells were resuspended at a ratio of 5 ml of lysis buffer per gram of pellet, using a buffer with 20 mM Tris-HCl pH 8.0, 150 mM NaCl and 10% (v/v) glycerol. Once resuspended, the suspension was supplemented with protease inhibitor tablets (typically, one for 100 ml of suspension of cOmplete, EDTA-free protease cocktail tablets, Merk) and DNases. A glass homogenizer was used for a good resuspension and first soft lysis step; 30 strokes were applied whilst keeping the homogenizer on ice. Finally, digitonin detergent (Merk) was used in cell lysis at a final concentration of 1%, and the suspension was incubated in rotation for at least one hour at +4 °C. Cell debris and non-solubilized material was directly removed by ultracentrifugation (Ti70 rotor, 165,442 *× g*, 1 h, +4 °C). In the meantime, the Ni-NTA agarose resin (QIAGEN) required for the purification was pre-equilibrated in wash buffer A (20 mM Tris-HCl pH 8.0 RT, 150 mM NaCl, 0.1% digitonin, 10% glycerol, 20 mM Imidazole). Ultracentrifugation supernatant was incubated with the resin for at least one hour in moderate rotation at +4 °C, and the affinity chromatography was performed on batch using gravity columns (Biorad). The resin was extensively washed with buffer A and additionally, with buffer B (same composition as buffer A but with increasing imidazole concentration, 30 mM) and C (40 mM imidazole). Finally, the protein was eluted with elution buffer, containing up to 150 mM imidazole.

The elution fraction was concentrated in a 100 kDa MWCO concentrator (Corning® Spin-X® UF concentrators) down to 100 μl and they were injected in a Superdex200 Increase 3.2/ 300 of 2.4 ml column volume, using a buffer without glycerol (20 mM Tris-HCl pH8.0, 150 mM NaCl and 0.1% digitonin). A monodispersed peak eluted at 1.3–1.4 ml and corresponded to the heterodimer Asc1/CD98hc, as analysis by SDS-PAGE confirmed. The concentration of the peak fractions (50 μl each) was measured and either pooled or kept separate depending on the concentration. In most attempts it was not required to concentrate the protein any further and grids were set up using 1.2–1.5 mg ml^−1^ of heterodimer and adding 10 mM D-Ser (Sigma) and incubating the sample of ice for at least 30 min. The purified heterodimer was applied to glow-discharged Cu Quantifoil 0.6/1 μm grids (Quantifoil) directly after size exclusion chromatography and vitrified using a FEI Vitrobot (Thermo Fisher Scientific), keeping the chamber at 100% humidity and +4 °C. A preliminary grid quality was assessed in an inhouse Tecnai T20 microscope (Thermo Fisher Scientific), guiding grid optimization.

### CryoEM data collection

Best grids were used for small data set collection at a JEOL JEM-2200FS microscope, equipped with a K3 camera (Gatan). The data set analysis was useful to determine particle homogeneity and heterodimer integrity. Large data collection was performed at a high-end Titan Krios (Thermo Fisher Scientific) 300 kV microscope (Leicester, UK) equipped with a K3 camera (Gatan) and operated remotely. For this, the peak fraction of the size exclusion chromatography was applied to holey grids at concentrations between 1.2 and 1.5 mg ml^−1^, vitrified and observed using a 300 kV Titan Krios electron microscope (Thermo Scientific™) equipped with a K3 camera. A total of 31,696 movies (50 frames) were collected in super-resolution mode using EPU. The set up used a 130 K magnification, which corresponded to 0.656 Å per pixel. The applied defocus covered a range from −0.8 to −2.4 μm, and the total electron dose was of 50 e^−^ Å^−2^.

### CryoEM image processing

Movies were imported in RELION4^40^, motion corrected using MotionCor^41^ and the CTF was estimated using CTFFIND-4.1 approach^42^. Moreover, a micrograph cleanup was done using RELION analyse^43^ to remove bad micrographs in terms of ice thickness and motion. A first particle picking was performed in a subset of 1000 micrographs randomly selected, manually picking ~1000 particles which were used as templates for training Topaz^44^. Finally, particle selection in the full data set was performed with Topaz and a total number of 2,699,978 particles were picked and extracted binned by 3 (1.95 Å per pix) setting the FOM at 1. Particles were then moved to cryoSPARC v3.2.0^45^ for a quick 2D classification, reducing particle number to 852,488. Particles were then moved back to RELION for iterative rounds of 3D reconstruction. A subset of 139,507 particles yielded a good initial model, so they were re-extracted at 0.831 Å per pix. Refinement by non-uniform refinement^30^ in cryoSPARC allowed micelle removal, and yielded a map with an overall map resolution of 4 Å. Maps were evaluated and analyzed using UCSF Chimera^46^ and map sharpening was tested with different programs to inspect different levels of details, among them LAFTER^47^ or phenix auto.sharpen^48^, but these maps were never used for further refinement.

### Model building

Model building was carried out in coot^49^ using the Asc1 AlphaFold model as starting point^31,32^. The model was modified according to the map density, except for some small regions corresponding to residues 54–57, 284–290 and 453–462 where the resolution of the density did not allow for an unambiguous modeling, and then the AlphaFold model was maintained. Model refinement and validation were performed in phenix^50^.

### Mutagenesis and transfection of WT hAsc1/CD98hc and mutants

HeLa cells (catalog number CCL-2, ATCC®) were transiently transfected in a 24-well plate with 250 ng per well of the human Myc-Tag-Asc-1 (N-terminally tagged) in pRK5 (kindly donated by Prof. Dr. Herman Wolosker, Technion – Israel Institute of Technology) and 250 ng per well of the Strep-TagII-CD98hc (isoform f) (N-terminally tagged) in pcDNA4-His-MaxC^33^, using GenJet^TM^ (SignaGen®, Ijamsville, MD). Single point mutations were introduced using the QuikChange mutagenesis kit (Stratagene) with the primers shown in Supplementary Data 1. All mutations were verified by sequencing.

### Cell-based uptake assays

Amino acid uptake measurements were performed on Asc1/CD98hc-transfected HeLa cells maintained at 37 °C in a humidified 5% CO_2_ environment in DMEM supplemented with 10% fetal bovine serum, 50 units ml^−1^ penicillin, 50 μg ml^−1^ streptomycin and 2 mM L-glutamine. For this purpose, HeLa cells were transiently transfected in a 24-well plate with 250 ng per well of the human Myc-Tag-Asc1 (N-terminally tagged) in pRK5 and 250 ng per well of the Strep-TagII-CD98hc (isoform f) (N-terminally tagged) in pcDNA4-His-MaxC, using GenJet^TM^ (SignaGen®, Ijamsville, MD). The experiment was performed 24 h after transfection. Uptake rates were measured as previously described^33^. Briefly, replicate cultures were incubated with 20 μM cold L-amino acid (glycine, L-alanine, L-serine, D-serine and L-valine, purchased from Merck, Darmstadt, Germany) and 1 μCi ml^−1^ of the tritiated amino acid ([2-^3^H]-glycine and L-[3-^3^H]-serine purchased from Perkin Elmer, Boston, MA; L-[2,3-^3^H]-alanine, D-[3-^3^H]-serine, and L-[2,3,4-^3^H]-valine purchased from American Radiolabeled Chemicals, St. Louis, MO) at room temperature for 1 min (linear conditions) in a sodium-free (137 mM choline chloride) transport buffer that also contained 5 mM KCl, 1.53 mM CaCl_2_, 507 μM MgSO_4_ and 10 mM HEPES (pH 7.4). Uptake was terminated by washing with an excess volume of chilled transport buffer. Transporter-mediated amino acid uptake was calculated by subtracting the uptake measured in mock-transfected cells. Transport was normalized by cell protein content determined by BCA assay. For kinetic studies, cells were incubated with 1 μCi ml^−1^ [^3^H] amino acid and varying concentrations of unlabeled amino acid (5–2000 μM). At high D-serine concentrations, 3 μCi ml^−1^ [^3^H] D-serine was used. The Michaelis-Menten and Eadie-Hofstee equations were then applied to calculate K_m_ and V_max_ values (Supplementary Fig. 10) using the GraphPad Prism software. Data are expressed as the mean ± s.e.m. of three experiments performed on different days and on different batches of cells.

### Amino acid efflux assays

Amino acid efflux measurements were performed on Asc1/CD98hc-transfected HeLa cells. D-[-3-^3^H]-serine was the substrate chosen for efflux experiments because of its higher transport V_max_ and lower rate of metabolism (vs other faster-metabolizing substrates such L-alanine), thus diminishing the experimental background of the experiment. Replicate cultures were incubated with 20 μM cold D-serine and 1 μCi ml^−1^ D-[-3-^3^H]-serine at room 37 °C for 5 min in a sodium-free (137 mM choline chloride) transport buffer that also contained 5 mM KCl, 1.53 mM CaCl_2_, 507 μM MgSO_4_ and 10 mM HEPES (pH 7.4). The cells were then washed 3 times with ice-chilled transport buffer and incubated for 5 min with transport buffer containing either 0.5% DMSO alone or together with 5 μM of BMS466442 (Tocris Bioscience, Bristol, United Kingdom) or 3 mM cold D-serine. Efflux was measured at the indicated times and is expressed as the percentage of the radioactivity measured in the transport buffer respect to the total amount loaded (efflux plus the remnant inside of the cells). Transporter-mediated amino acid efflux was calculated by subtracting the efflux measured in cells treated with the Asc-1 specific inhibitor BMS466442. Data are expressed as the mean ± s.e.m. of three to five experiments performed on different days and on different batches of cells.

### Time difference estimation for out-to-in transition in hAsc1

We designed the following model to estimate the effect of mutations of Asc1/CD98hc in each mode of transport described in Fig. 4 and Supplementary Fig. 11. We considered the time to complete the efflux of 1% of the loaded radioactivity (i.e., the inverse of the Efflux shown in Fig. 4, represented in Supplementary Fig. 11g) as follows:

A. Time for efflux by Exchange (t_EFFLUX_E) = t_OUT_ + t_IN_E; where t_IN_E is the time to return the transporter bound to substrate (cold D-serine) to face the cytosol (blue arrows in Supplementary Fig. 11a).
B. Time for efflux by diffusion (t_EFFLUX_D) = t_OUT_ + t_IN_D; where t_IN_D is the time to return the transporter empty (efflux by diffusion) to face the cytosol (dashed arrows in Supplementary Fig. 11a).

In both cases, t_OUT_ is the time to transport substrate radioactivity to the extracellular medium, which is identical in efflux by exchange and efflux by diffusion conditions because is a common step (red arrows in Supplementary Fig. 11a). Therefore, t_EFFLUX_D – t_EFFLUX_E = t_IN_D-t_IN_E.

t_IN_D-t_IN_E is the difference between the time necessary for the return of the outward to inward transition empty (diffusion) or bound to substrate (exchange) necessary to complete the efflux of 1% of the loaded radioactivity.

### Intracellular amino acid quantification

Frozen pellets from HeLa cells were resuspended in 200 µl of phosphate-buffered saline. Cells were lysed by sonication, using cycles of 10 s (40% amplitude, on ice). Samples were frozen at −80 °C until analysis. Samples were thawed and centrifuged for 10 min at 10 °C at 600 × *g*. Amino acid levels were measured in the supernatants by UPLC-MS/MS, as previously described^51^. Briefly, 10 µl of the cell supernatants were mixed with 25 µl of the internal standard solution (a mix of 17 amino acids labeled by isotopes 13 C and 15 N; Cambridge Isotope Laboratories, Inc. REF MSK-A2-S) and 40 µl of methanol/0.1% formic acid to precipitate the proteins. Then, samples were centrifuged at 600 × *g* for 10 min at 10 °C. For the derivatization reaction, 5 µl of the supernatant was mixed with 35 µl of borate buffer and 10 µl of AQC solution (3 mg ml^−1^ in acetonitrile; AccQ·Tag Ultra Derivatization Kit, REF 186003836). The samples were analysed using a Waters ACQUITY UPLC H-class coupled to a Waters Xevo TQD triple-quadrupole mass spectrometer using positive electrospray ionization in the multiple reaction monitoring mode. Quantification was normalized by total protein by BCA assay.

### Immunocytochemistry

HeLa cells were seeded on 12 mm circular glass coverslips and transiently co-transfected with Asc1/CD98hc as previously described. 24 h after transfection the slides were washed with PBS and then incubated with 2.5 μg ml^−1^ of Texas Red-X-labeled wheat germ agglutinin (Thermo Fisher Scientific) at room temperature for 10 min. The cells were subsequently washed with PBS and fixed with 4% paraformaldehyde in PBS at room temperature for 20 min. The fixed cells were then permeabilized with a solution of 0.1% Triton X-100, 1% BSA in PBS at room temperature for 30 min. Afterwards the coverslips were incubated with a 1:500 dilution of anti-Myc-tag antibody (05–724, Millipore) in 1% BSA in PBS at room temperature for 1 h, followed by washes with PBS and a second incubation with a secondary Alexa Flour 488-labeled anti-mouse IgG antibody (A11029, Thermo Fisher Scientific) in 1% BSA in PBS at room temperature for 1 h. Nuclear staining was performed with a 1:20,000 dilution of Hoechst 33342 (Thermo Fisher Scientific) in PBS at room temperature for 20 min. Coverslips were then mounted using Fluoromount^TM^ aqueous mounting medium (Thermo Fisher Scientific) and imaged using a Leica TCS SP5 Spectral Confocal Multiphoton system.

### Immunoblotting

HeLa cells were seeded on 6-well plates and transiently co-transfected with Asc1/CD98hc as previously described. 24 h after transfection, the cells were washed with PBS and then gently scraped with a Corning® Cell Lifter. The pelleted cells were lysed in buffer containing 20 mM Tris pH 8, 150 mM NaCl, 10% glycerol, 1% IGEPAL CA-630 and cOmplete^TM^ Mini protease inhibitors for 20 min on ice. The lysates were then centrifuged at 4 °C for 15 min at 15,000 × *g*. Protein concentration in the supernatant was determined using the bicinchoninic acid (BCA) assay. Separation of proteins by molecular weight was carried out by SDS-PAGE on 12.5% polyacrylamide gels, loading 25 μg of total protein per sample.

For Western Blot analysis, the resolved proteins were transferred to Immobiolon®-FL PVDF membranes and incubated with primary antibody. Anti-Myc-tag mouse monoclonal antibody (catalog number 05–724, clone 4A6, Millipore) and anti-SERCA2 rabbit monoclonal antibody (catalog number 9580, clone D51B11, Cell Signaling Technology®) were used in a 1:1000 dilution. For detection fluorescent secondary antibodies were used. Goat anti-Mouse IgG (H + L) secondary antibody - DyLight 680 (catalog number 35518, Thermo Fisher Scientific), goat anti-Mouse IgG (H + L) secondary antibody - DyLight 800 4X PEG (catalog number SA5-35521, Thermo Fisher Scientific), goat anti-Rabbit IgG (H + L) secondary antibody - DyLight 680 (catalog number 35568, Thermo Fisher Scientific) and goat anti-Rabbit IgG (H + L) secondary antibody - DyLight 800 4X PEG (catalog number SA5-35571, Thermo Fisher Scientific) were used at 1:10000 dilution. The blots were developed using a LiCor Odyssey Infrared Imaging System 9120.

### Statistical analysis for transport studies

In the efflux experiments, efflux percentage was divided by the acquisition time to obtain rates of efflux per minute. Technical replicates were summarized through the mean and the inverse was computed to obtain a measure of minutes per unit of efflux. A Boxcox transformation with lambda = 1.15 was applied to the resulting values and a linear model was fitted with the combination of measure type (Exchange or Diffusion) and genotype as covariable. The “glht” function from the multcomp R^52,53^ package was used to compute coefficients and *p* values of the comparisons of interest. In the uptake experiments, data normalized for glycine uptake was transformed using a Boxcox transformation with parameter 0.05. A linear model was fitted to this data with condition and experiment as covariable. The glht function was used to find coefficients and *p* values of the contrasts of interest with no internal *p* value adjustment. *p* value correction was performed a posteriori with the Benjamini-Hochberg method. The uptake experiments normalized with L-serine uptake were analyzed with a similar methodology except that it was transformed with the logarithm. For the analysis of kinetics data, the same type of transformations and models were used. For the K_m_ parameter, the boxcox transformation was done with alpha = 0.05 and alpha = 0 (logarithm) for V_max_.

### PELE enzyme-substrate interaction modeling

The cryoEM structure of hAsc1 was prepared for PELE simulations with the Protein Preparation Wizard (PrepWizard) tool implemented in Schrödinger^54^. PROPKA 3.0 was used to calculate the protonation state of titratable residues at pH 7.4 and, based on the predicted pKa values, the hydrogen-bonding network was optimized. The resulting structure was subjected to a restrained minimization step with the all-atom optimized potentials for liquid simulations (OPLS-AA) force field (FF), keeping heavy atoms in place and optimizing only the positions of the hydrogen atoms.

The PELE software was used to map the enzyme-substrate interaction^35^. PELE is a heuristic Monte Carlo (MC) procedure designed to map protein–ligand induced fit interactions and extensively used in drug design^55^ and enzyme engineering^56^. Each MC step involves a complex series of events, including ligand and protein (backbone) perturbation, side-chain sampling, and a minimization. Typically, tens to hundreds of thousands of MC steps are used to explore the substrate binding (or migration) space, where we record structural parameters and the OPLS2005 FF enzyme-substrate interaction energy. Two sets of simulations were performed. First, from an initially docked structure obtained with the Glide software^57^, we ran a local PELE exploration for each substrate in the wild-type transporter. As expected, PELE retrieved the canonical pose as the main minimum, which could not be obtained in the initial docking due to the closeness of the apo form. The second set, started from the canonical pose, and the ligand was allowed to explore a larger space; the center of mass was allowed to move within an 8-Å window. For each enzyme-substrate system, simulations involved 128 computing cores running for 1250 MC steps each, which involved on average ~36 wall clock hours on the MareNostrum IV supercomputer at the Barcelona Supercomputing Center.

### Molecular dynamics

The cryoEM structure of Asc1 bound to L-alanine according with the pose with the lower binding energy (Fig. 3b) was prepared for molecular dynamics simulations with PrepWizard tool^54^. Missing hydrogen atoms were then added by the utility apply applythreat in the PrepWizard tool. PROPKA 3.0 was used to calculate the protonation state of titratable residues at pH 7.4 and, on the basis of the predicted pK values, the hydrogen bonding network was optimized. The resulting structure was subjected to a restrained minimization step with the OPLSAA force field (FF), keeping heavy atoms in place and optimizing only the positions of the hydrogen atoms.

To model the membrane in the system, Asc1 coordinates were pre-oriented with respect to the membrane (parallel to the z-axis) by alignment with AdiC (PDB 3OB6) in the OPM database (http://opm.phar.umich.edu)^58^. The protein was then embedded in a POPC lipid bilayer using the CHARMM-GUI Membrane Builder^59–62^ by the replacement method. Next, 150 lipid molecules were placed in the lipid bilayer (i.e., 75 lipids in each leaflet) with its center at z = 0. The system was then solvated using a TIP3PM water layer of 20 Å thickness above and below the lipid bilayer. NaCl ions corresponding to 0.15 M (29 negative and 35 positive) were also added to the system using Monte Carlo sampling.

In the case of ligand L-alanine, the automated ligand FF generation procedure (CGenFF) available in CHARMM-GUI was used to generate the FF parameters. Finally, with the CHARMM-GUI Membrane Builder, we also generated the necessary scripts to perform minimization, equilibration and production runs in AMBER, using the CHARMM36 force field (C36 FF), as explained below.

We ran three replicas with different sets of randomly generated initial velocities, using the C36 FF for lipids and the CHARMM TIP3P water model, at constant temperature (300 K) and pressure (1 bar), under Periodic Boundary Conditions, and with Particle Mesh Ewald electrostatics. The simulation time step was set to 2 fs in conjunction with the SHAKE algorithm to constrain the covalent bonds involving hydrogen atoms. After standard Membrane Builder minimization (2.5 ps) and equilibration (375 ps in 6 steps), production simulations were run with 500 ns per trajectory.

### Reporting summary

Further information on research design is available in the Nature Portfolio Reporting Summary linked to this article.

### Supplementary information

Supplementary Information

Peer Review File

Description of Additional Supplementary Files

Supplementary Data 1

Supplementary Data 2

Reporting Summary

### Source data

Source Data

## Data Availability

The cryo-EM map has been deposited in the Electron Microscopy Data Bank (EMDB) under the accession code EMD-18379 (apo human Asc1/CD98hc in inward-facing semi-occluded conformation). The atomic coordinates have been deposited in the Protein Data Bank (PDB) under the accession code 8QEY (apo human Asc1/CD98hc in inward-facing semi-occluded conformation). The previously-published atomic coordinates referred to in the text, and shown in Fig. 2, are available in the Protein Data Bank (PDB) under the accession codes 7B00 (apo human LAT2/CD98hc in inward-facing open conformation) and 7DSQ (3,5-diiodo-L-tyrosine-bound human LAT1/CD98hc in outward-facing occluded conformation). Molecular dynamics trajectories (3 replicas for apo Asc1 and 3 replicas for holo L-alanine bound-Asc1) as well as PELE raw data (initial dockings for L-alanine, L- and D-serine, the final energy minimums shown in the paper, the.conf files needed to run PELE and the run file used to queue the calculations at the MareNostrum supercomputer) have been deposited in a Zenodo repository [10.5281/zenodo.10788789]. For molecular dynamics studies, the Asc1 coordinates were pre-oriented with respect to the membrane by alignment with AdiC (PDB 3OB6) (Structure of AdiC in the open-to-out Arg^+^ bound conformation). Primer sequences used for mutagenesis studies are provided in the Supplementary Data 1 file. The source data underlying Figs. 3c-4 and Tables 1–2 are provided as a Source Data File; and Supplementary Figs. 1b, 8e, f, 9c, 11e, f, g, h and 15 are provided in the Supplementary Data 2 file. All other data generated in this study is available within the Supplementary Information file. Source data are provided with this paper.
