# Supplementary material for: Structure and mechanisms of transport of human Asc1/CD98hc amino acid transporter
Source: Nat Commun. 2024 Apr 6;15:2986. doi: 10.1038/s41467-024-47385-3 (PMC10998858; doi:10.1038/s41467-024-47385-3)

Supplementary Figures

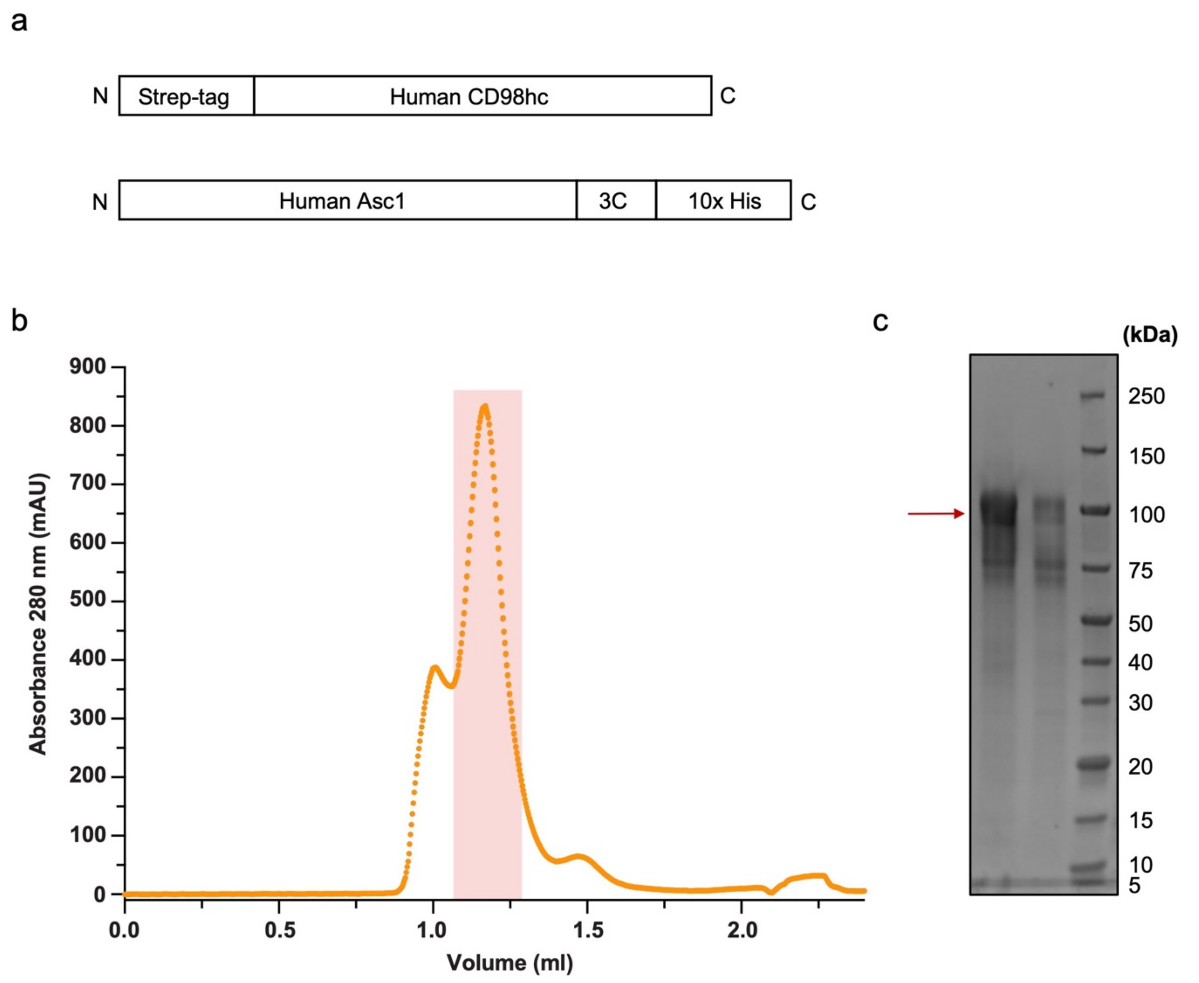

Supplementary Figure 1. Asc1/CD98hc construct design and purification. a, Asc1/CD98hc was expressed in HEK cells grown in suspension, co-transfected with two independent plasmids: one encoding for the heavy chain CD98hc and a second, for Asc1 light chain. CD98hc carries a Strep-tag at N-terminus, whereas Asc1 has a 6xHis-tag at C-terminus preceded by a PreScission (3C) cleavage site. **b,** Size exclusion chromatogram (SEC) for Asc1/CD98hc as a final purification step, using a Superose6 increase column 3.2/300 (2.4 ml). The main peak (red box) was fractionated in 50 µl fractions, which were directly used for sample vitrification. **c,** SDS-PAGE sample analysis of the two main peak fractions of the SEC run. Asc1/CD98hc runs at >100 kDa as indicated by the red arrow. Sample contaminant Hsp70 runs at ~70 kDa.

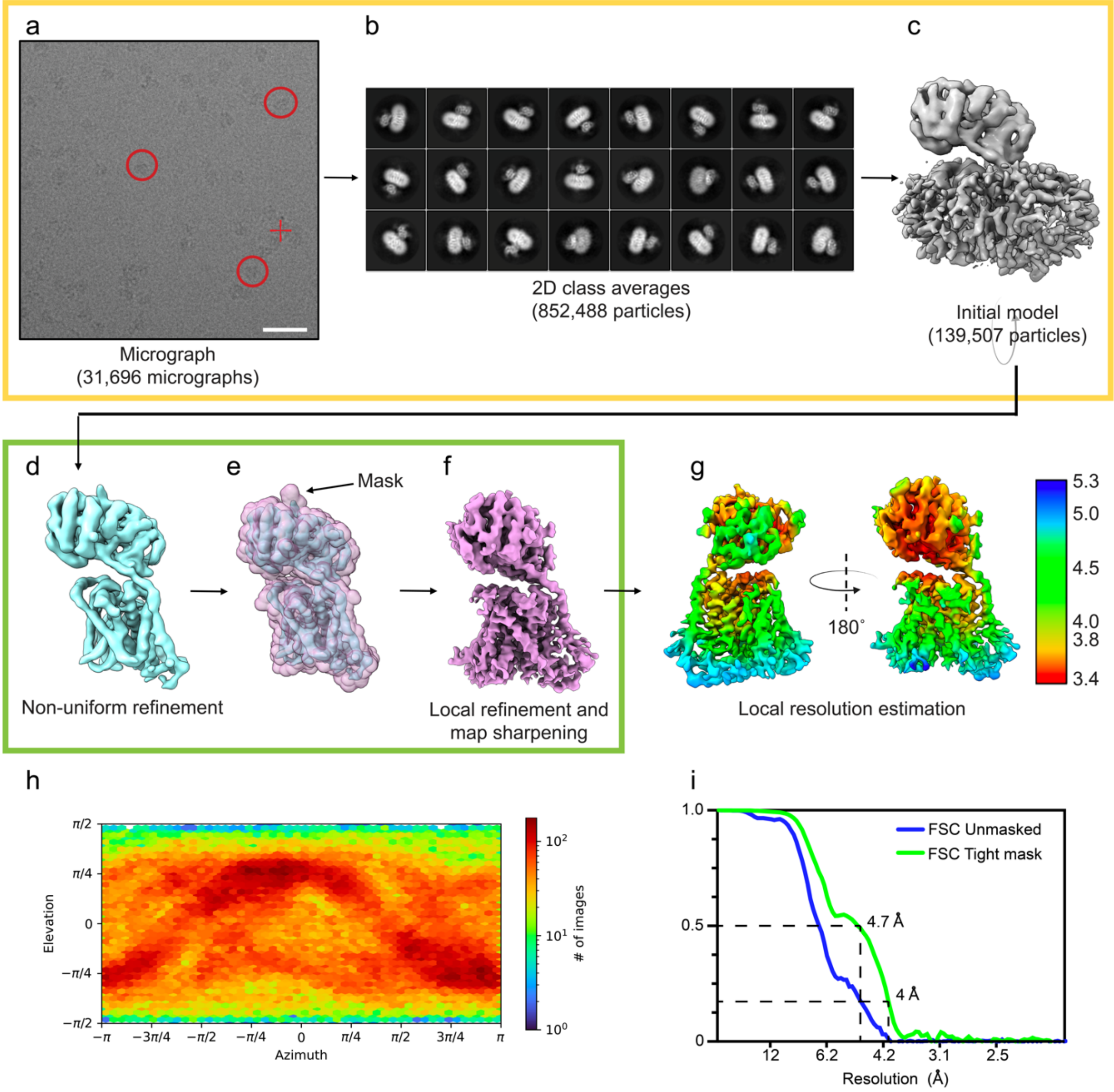

Supplementary Figure 2. CryoEM processing workflow. a, Representative micrograph, where the scale bar corresponds to 50 nm and the red circles surround individual Asc1/CD98hc particles. **b,** Representative 2D class-averages. **c,** Initial 3D reconstruction. The grey arrow indicates that this step was repeated iteratively to remove bad particles. **d,** 3D map obtained by non-uniform refinement. **e,** Map shown in panel d with the tight mask used in the final local refinement. **f,** Locally refined sharpened map once the micelle noise contribution was removed. **g,** Local resolution map color-coded according to the resolution estimation. **h,** Viewing direction distribution plot. **i,** Fourier shell correlation (FSC) plot of local refinement with FSC thresholds at 0.143 and 0.5. In panels a to f, data processing carried out in RELION is surrounded by a yellow square, whereas those parts of analysis done in cryoSPARC are surrounded in green.

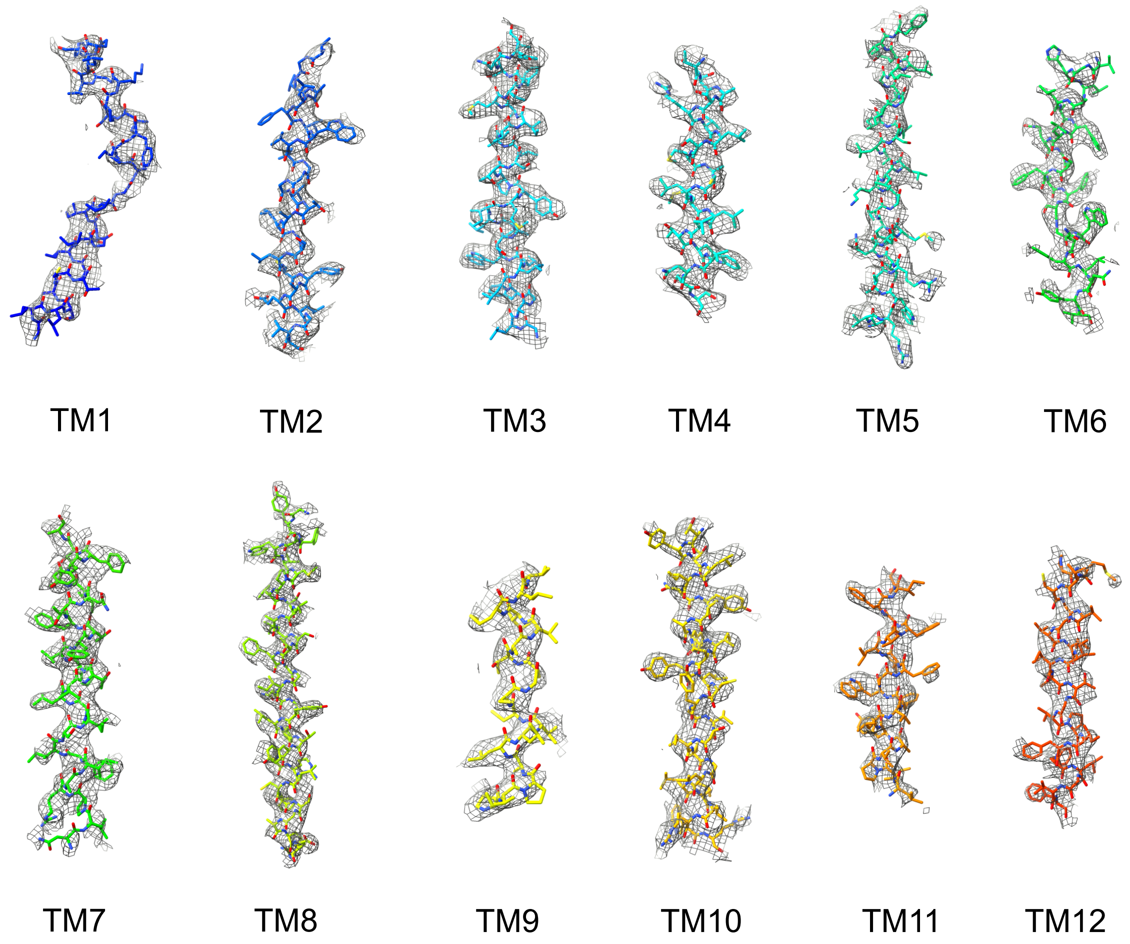

Supplementary Figure 3. Details of the cryoEM map for Asc1. Density corresponding to the individual Asc1 TM helices with the corresponding model fitted inside the density. C atoms are coloured according with the colour of the corresponding TMs as in **Fig. 1**.

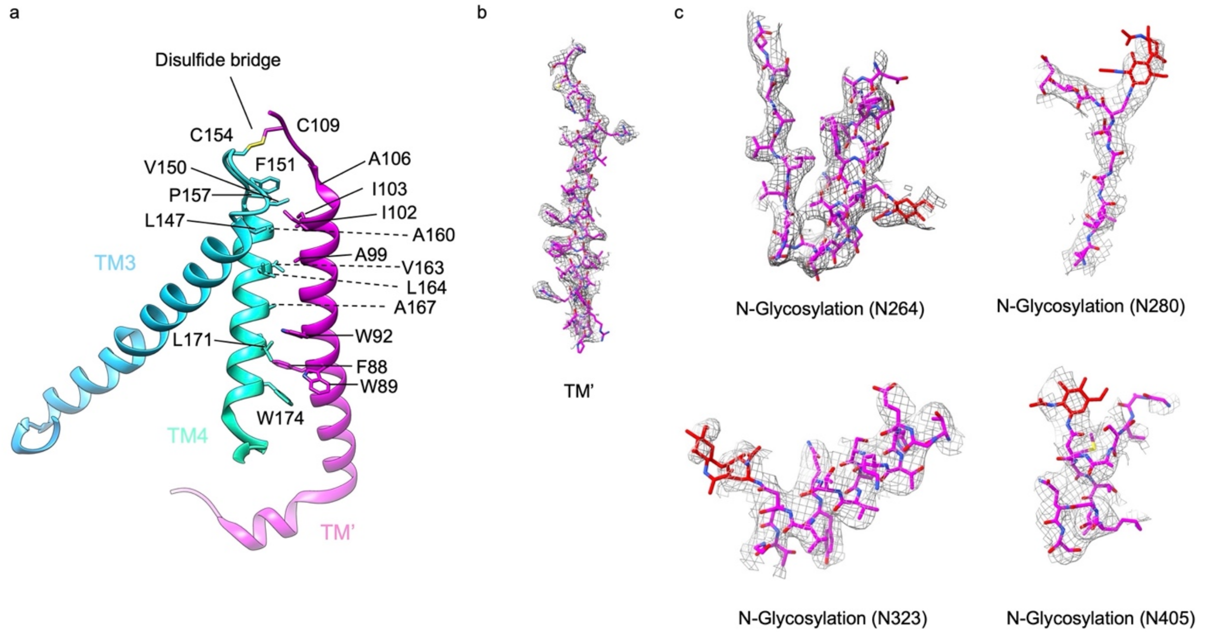

Supplementary Figure 4. Structural details and glycosylation sites for CD98hc. a, CD98hc transmembrane helix (TM') model, and interactions with TM3 and TM4 of Asc1. C atoms and helices follow the colour-code of **Fig. 1**. **b,** CryoEM density for TM' in CD98hc and the fitted atomic model. **c,** detail of N-glycosylations in the soluble ectodomain in CD98hc, comparing the cryoEM density and the atomic model.

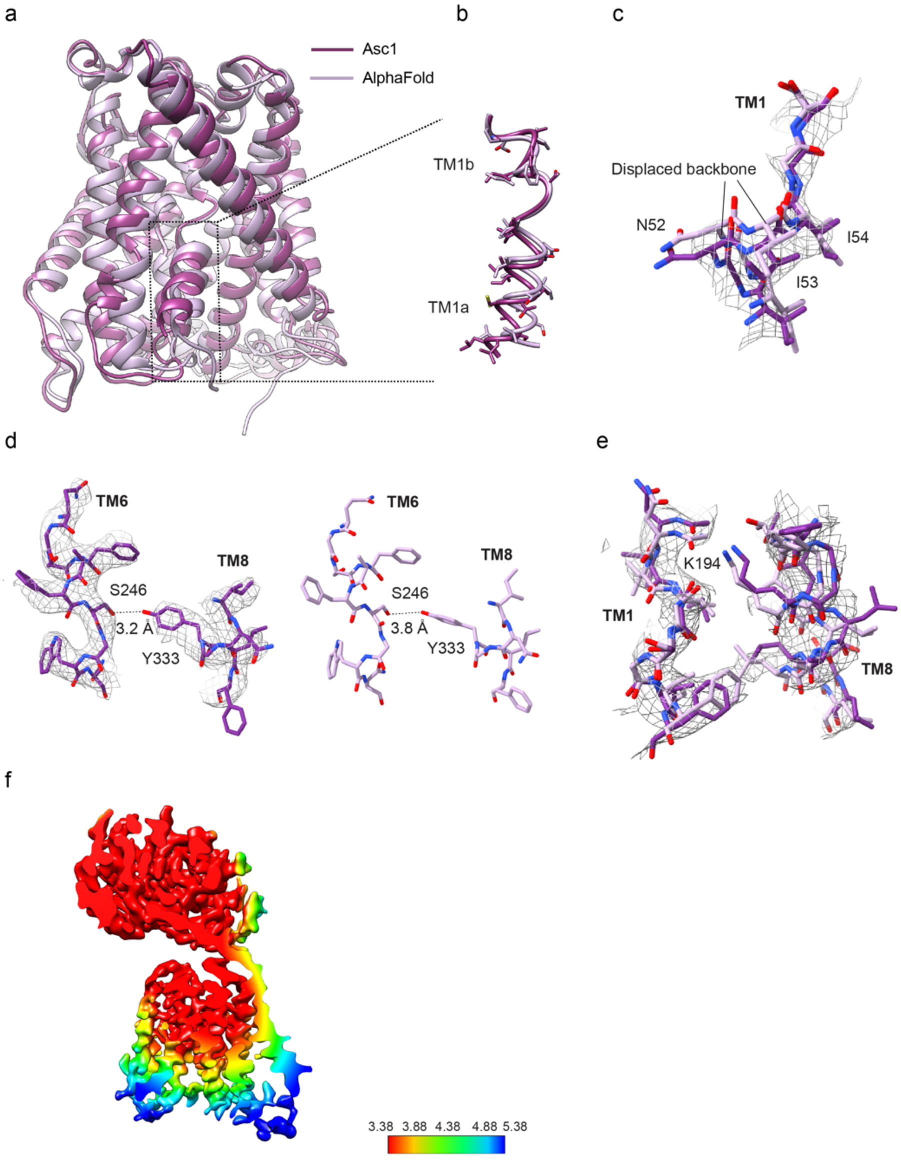

Supplementary Figure 5. Comparison between the experimental model of Asc1 (dark purple) and the AlphaFold prediction (light purple). a, whole structure. **b**, TM1, where our experimental model shows a semi-occluded conformation whereas the AlphaFold model predicts a fully closed TM1a. **c**, Close-up of residue Ile 53 in the TM1a, comparing the experimental model and the AlphaFold prediction. The AlphaFold prediction does not fit the density, and our atomic model was modified to fit the cryoEM density. **d**, Close-up for Y333-S246 hydrogen bond (dashed line) with the corresponding cryoEM density map of the area. Left panel shows the experimental cryoEM map fitted with the atomic model derived from our data. Right panel shows the same region in the AlphaFold prediction. **e**, Close-up of Lys 194 in TM5 that shows no density for the side chain in the cryoEM density, suggesting some flexibility. On the contrary, surrounding residues in TM1 show unambiguous density. **f**, Local resolution map colour-coded according to the resolution estimation as in Supplementary Figure 2, but cropped to highlight differences in resolution around TM1. The color-code scale is shown as a right-hand panel.

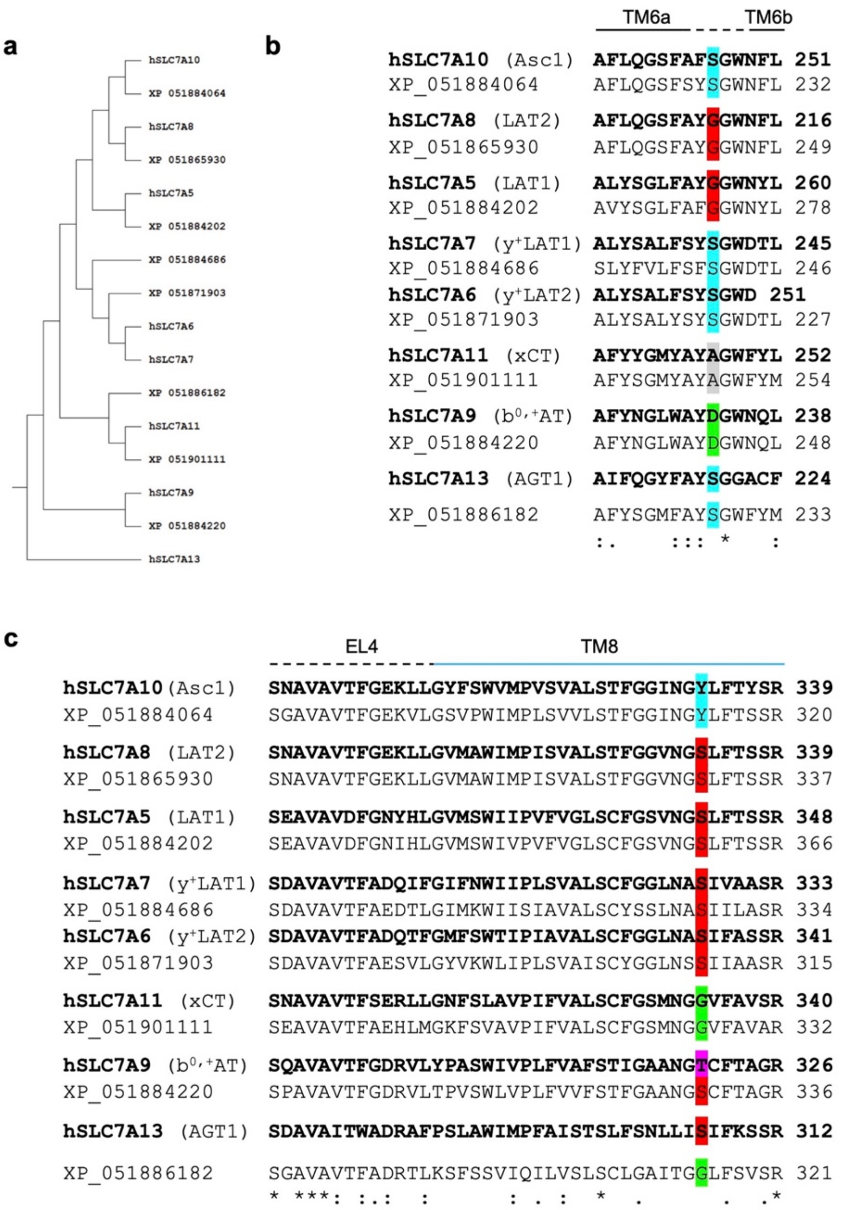

Supplementary Figure 6. Multialignment of human and smalltooth sawfish LAT sequences. **a**, Phylogenetic tree of the LAT sequences from smalltooth saw fish (*Pristis pectinata*) retrieved by BLAST with human SLC7A10 (Asc1) protein sequence. The phylogenetic tree was constructed using CLUSTALW in BioEdit to align the human LATs with the proposed LAT sequences of *Pristis pectinata*. The tree was produced using MEGA X employing the unweighted pair group method with arithmetic mean clustering. **b**, Multialignment showing that the Ser residue located in TM6 unwound segment (doted line) is conserved in human and smalltooth sawfish Asc1 sequences. This Ser residue is also present in y$^+$LAT1, y$^+$LAT2 and AGT1 sequences. **c**, Multialignment showing the specific Tyr residue in TM8 of human and smalltooth sawfish Asc1 sequences.

a

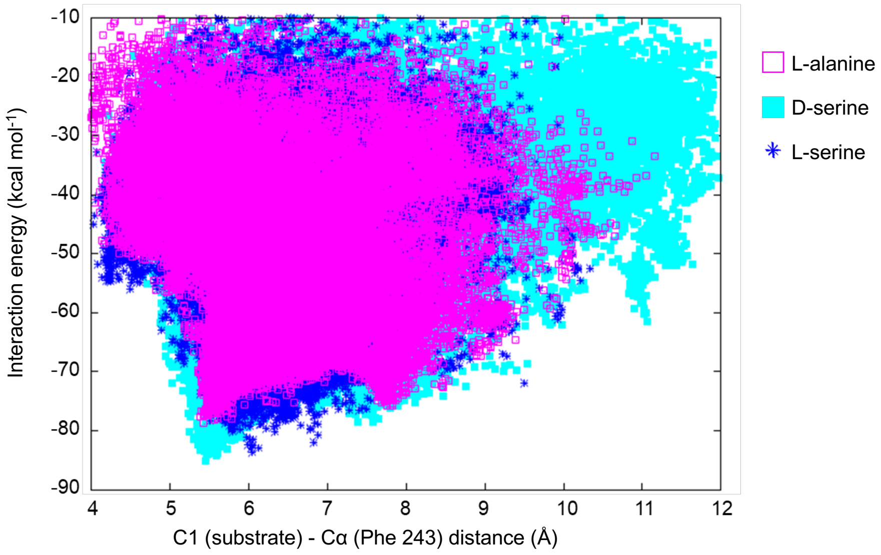

b

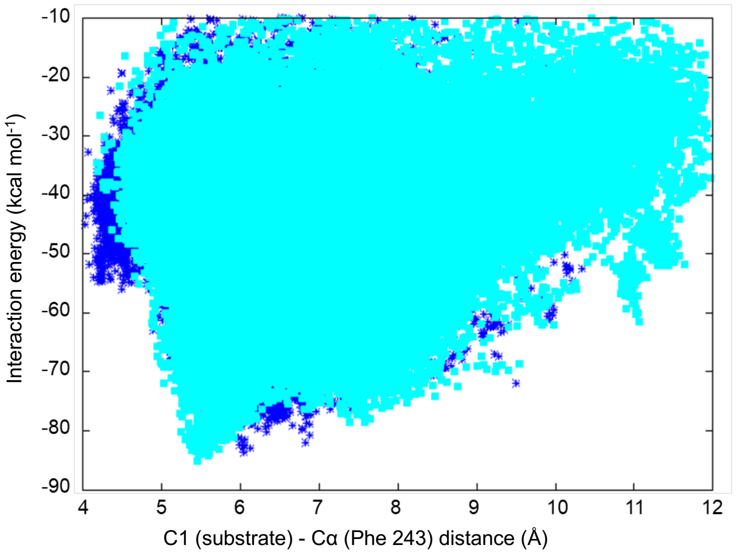

Supplementary Figure 7. Energy landscape of substrate binding to inward-facing semi-occluded Asc1. a, Results from PELE simulations displaying interaction energies for the amino acid substrates L-alanine (magenta), L-serine (dark blue) and D-serine (cyan) against the distance between alpha carbons of the substrate and of residue Phe 243. **b,** For clarity also D- and L-serine data alone are shown. L-alanine showed two minimma of binding-energy: pose 1 (**Fig. 3b**) corresponds to the minimmum at 5.4 Å of the indicated distance with a calculated binding-energy of -78 kcal mol^{-1}, and pose 2 (**Supplementary Fig. 8a**) corresponds to the minimmum at ~7.7 Å of the indicated distance with a calculated binding energy of -73 kcal mol^{-1}. L-serine showed two minimma of binding energy: poses 1 and 2 (**Supplementary Figures 8c and d**, respectively) correspond to the minimmum at 6 and 6.8 Å of the indicated distances and with calculated binding-energies of -83 and -82 kcal mol^{-1}, respectively. A single minimum for D-serine was obtained at 5.4 Å of the indicated distance with a calcuated binding energy of -85 kcal mol^{-1}. Simulations comprised 160,000 Monte Carlo PELE steps were attempted (~55,000 sampled binding modes) per substrate.

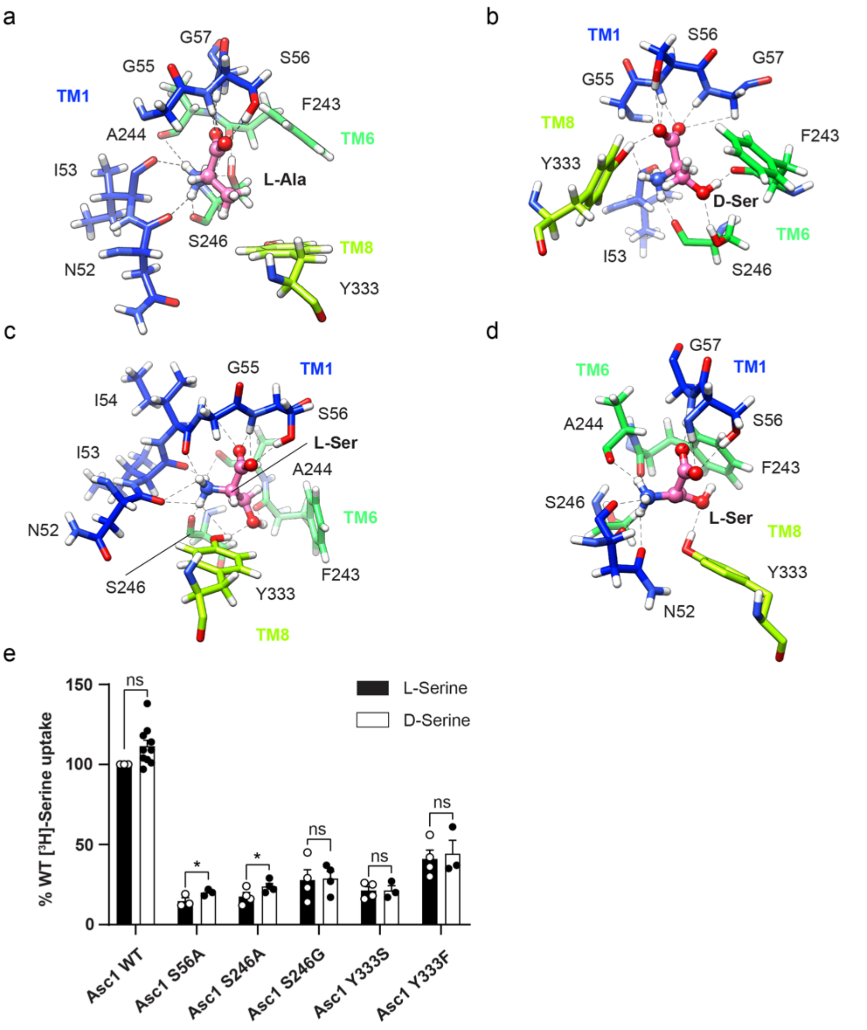

f

	D-Serine		L-Serine	
	K_m (μM)	V_{max} (pmol mg^{-1} protein min^{-1})	K_m (μM)	V_{max} (pmol mg^{-1} protein min^{-1})
hAsc1 WT	144 ± 14.8	13791 ± 2542	130 ± 20.6	10158 ± 1201
hAsc1 S246G	209 ± 9.6 (**)	4206 ± 95.3 (***)	-	-
hAsc1 Y333F	131 ± 10.2 (ns)	4059 ± 323.2 (***)	-	-

Supplementary Figure 8. The substrate binding site for L-alanine L- and D-serine. a, Second higher-energy pose for L-alanine obatined by PELE. **b**, Pose for D-serine predicetd by PELE. **c-d**, Two poses for L-serine obtained by PELE. Putative H-bonds are indicated by a dashed line. Substrate C and H atoms are coloured pink and white respectively. C atoms in the Asc1 residues are coloured according with the colour of the corresponding TM domains as in **Fig. 1**. N and O atoms are coloured blue and red respectively. **e**, Uptake 20 µM of the indicated amino acids in HeLa cells co-transfected with CD98hc and indicated Asc1 variants (WT and mutants S56A, S246A, S246G, Y333S and Y333F). Uptake activity induced by Asc1 is normalized for L-serine uptake mediated by WT Asc1/CD98hc (2371 ± 358 pmol mg^{-1} protein min^{-1} from 11 independent experiments). Data (mean ± s.e.m.) corresponds to n=3 (S56A, Y333S/F D-Ser), n=4 (S246A, S246G, Y333S/F L-Ser) or n=10 (WT D-

Ser) independent experiments. P-values were obtained by fitting a linear model to Boxcox transformed data (see materials and methods). P-values were adjusted for multiple comparisons using the Benjamini-Hochberg method: ns, non-significant; and (*), $p < 0.05$. WT L vs D-serine uptake, $p = 0.309$; S56A L vs D-serine uptake, $p = 0.036$; S246A L vs D-serine uptake, $p = 0.022$; S246G L vs D-serine uptake, $p = 0.949$; Y333S L vs D-serine uptake, $p = 0.681$; Y333F L vs D-serine uptake, $p = 0.778$. **f**, Kinetics analysis of the uptake of D- and L-serine for the indicated Asc1 variants. Data (mean ± s.e.m.) correspond to n=3 independent experiments run in triplicates. P-values were obtained by fitting a linear model to Boxcox transformed data (see materials and methods). A linear model was used to compare conditions after log-transforming the data. P-values were adjusted for multiple comparisons using the Benjamini-Hochberg method: n.s., non-significant; (**), $p \leq 0.01$; and (***), $p \leq 0.001$. WT vs S246G K_m, $p = 1.39 \cdot 10^{-3}$; WT vs S246G V_{max}, $p = 2.61 \cdot 10^{-7}$; WT vs Y333F K_m, $p = 0.365$; WT vs Y333F V_{max}, $p = 1.82 \cdot 10^{-7}$.

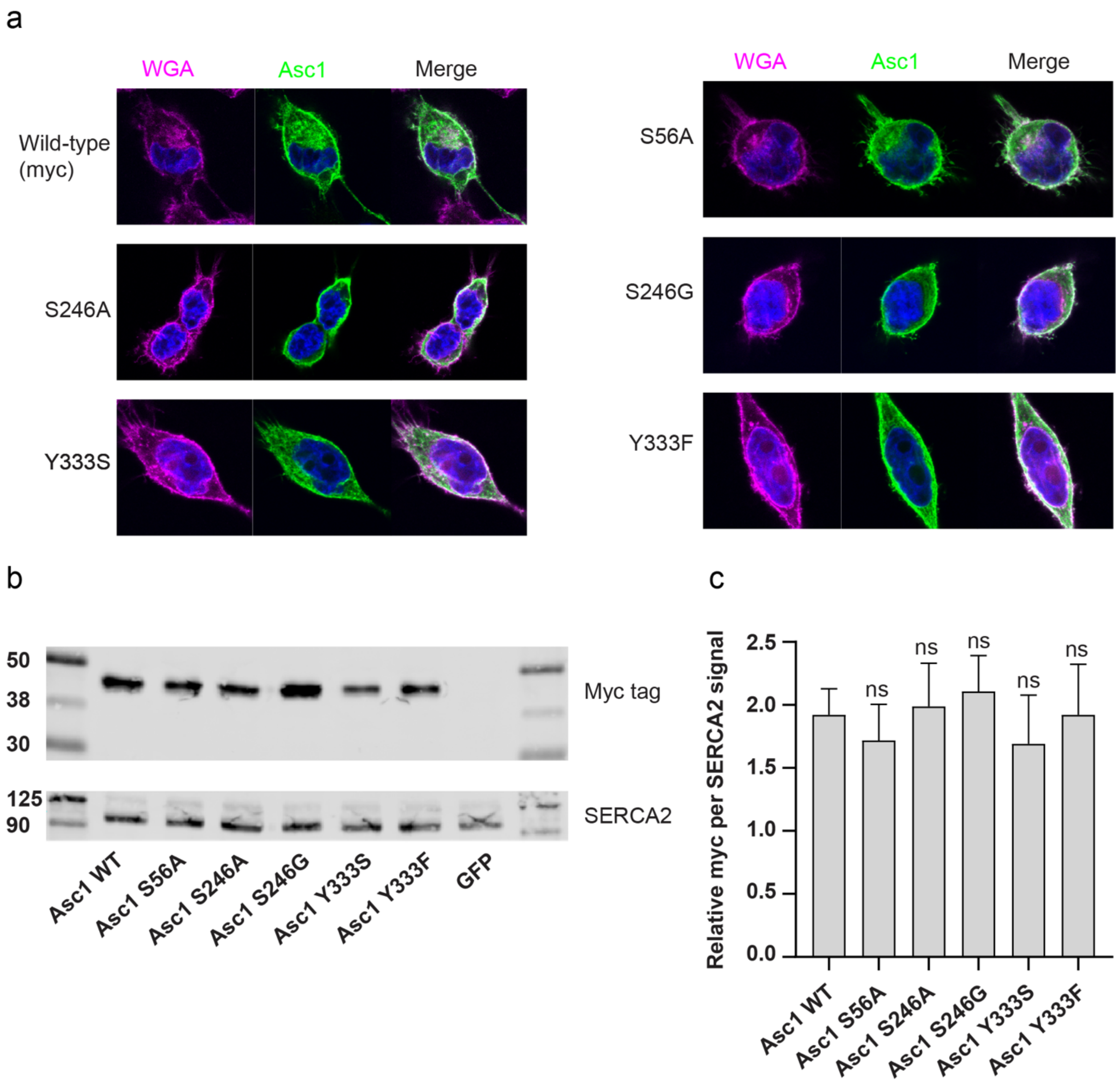

Supplementary Figure 9. Expression of human Asc1 mutants in HeLa cells co-transfected with CD98hc. **a**, Panel showing representative images of immunofluorescence of wild type and Asc1 mutants. C-terminal tagged with myc Asc1 (green), wheat germ agglutinin (WGA, membrane marker) (purple) and Hoechst 33342 (blue) labelling is shown. All Asc1 variants reached the plasma membrane. **b**, Representative Western blot of Asc1 detected with anti-myc (WT and S56A, S246A, S246G, Y333F and Y333S) normalised to endogenous SERCA2 expression. **c**, Quantification of Asc1 expression (mean ± s.e.m.) for n=5 (WT, S56A, Y333F), n=4 (S246G, Y333S) or n=3 (S246A) independent experiments. P-values were obtained by two-tailed Student's t test (see materials and methods). WT vs S56A, p = 0.580; WT vs S246A, p = 0.866; WT vs S246G, p = 0.604; WT vs Y333S, p = 0.594; WT vs Y333F, p = 0.999.

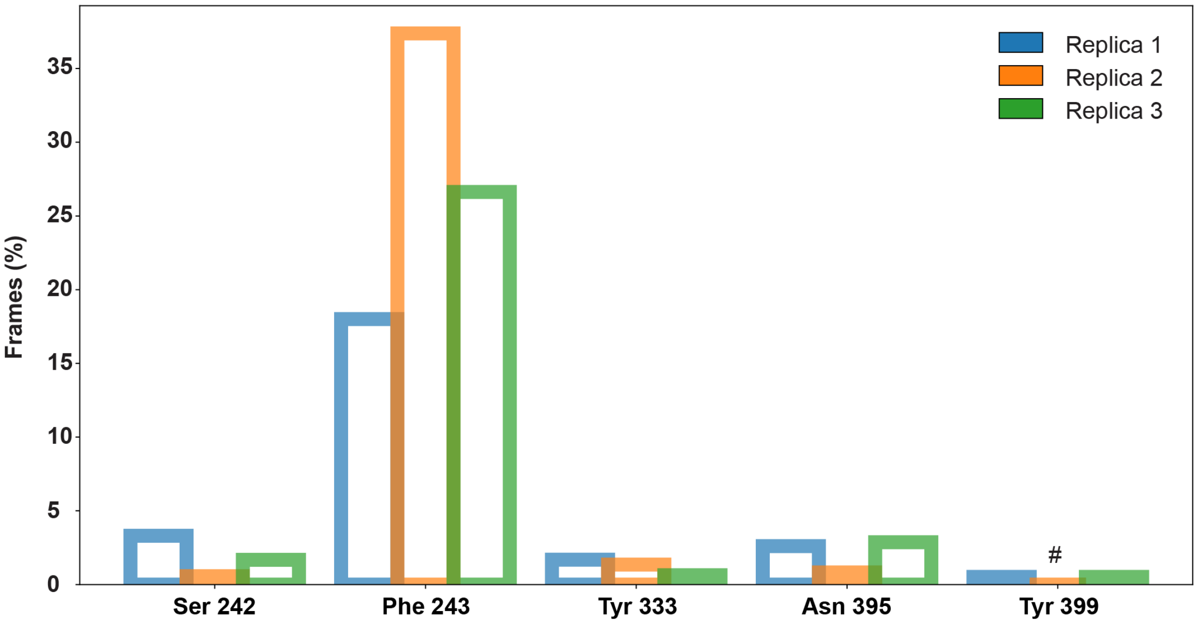

Supplementary Figure 10. Ser 246 hydroxyl group interactors. Molecular dynamics analysis of Asc1 apo structure (PDB ID 8QEY). Percentage of hydrogen bonds every 0.02 ns between the Ser 246 hydroxyl group and other residues along along three different replicas of 500 ns (blue, orange and green). All the interactions with residues that had < 0.5% of presence were considered residual and not plotted. #, interaction with <0.5% of presence shown for clarity.

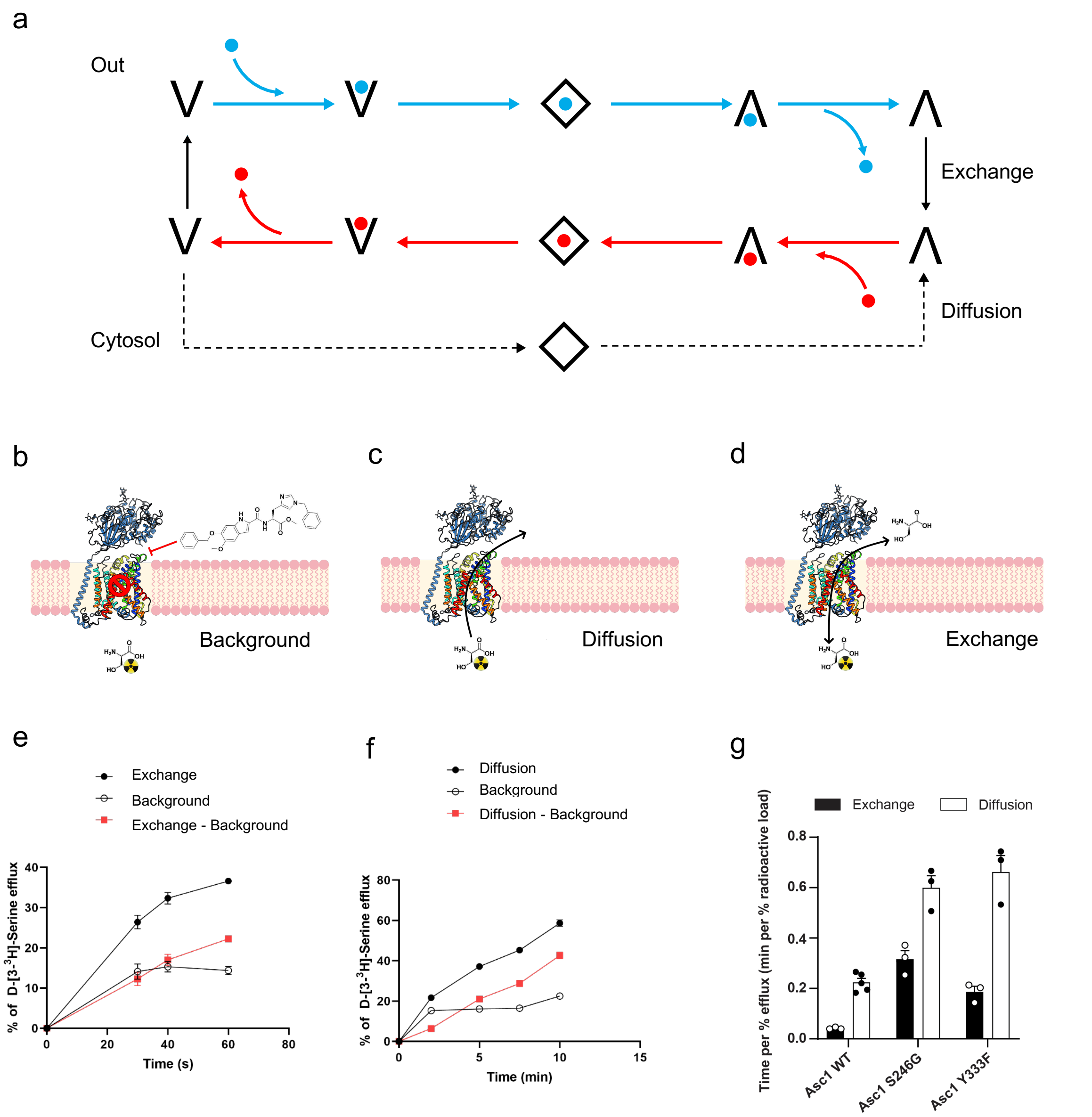

h

	Glycine	Alanine	Serine	Threonine	Total substrates	Total non-substrates
	Intracellular amino acid content (nmol amino acid mg^{-1} protein)					
hAsc1 WT	38.07 ± 0.9	17.01 ± 2.4	6.60 ± 0.1	18.20 ± 1.1	79.88 ± 3.2	143.60 ± 8.8
hAsc1 S246G	36.96 ± 2.4 (ns)	16.30 ± 1.1 (ns)	5.5 ± 0.1 (***)	21.65 ± 1.3 (ns)	79.97 ± 4.6 (ns)	139.05 ± 3.1 (ns)
hAsc1 Y333F	41.35 ± 2.8 (ns)	19.99 ± 3.8 (ns)	6.48 ± 0.4 (ns)	22.24 ± 2.8 (ns)	90.05 ± 8.3 (ns)	153.76 ± 13.8 (ns)

Supplementary Figure 11. Cell-based exchange or facilitated diffusion assays in Asc1/CD98hc co-transfected HeLa cells. a, Schematic representation of exchange or facilitated diffusion by hAsc1/CD98hc. In the diagram, Asc1 is represented in outward-facing (V), occluded (diamond shape) and inward-facing conformations (inverted V). The developed assay to measure efflux through facilitated diffusion or exchange involves measurements in three different conditions: **b**, Determination

of non-specific background by inhibition of Asc-1 with BMS466442. **c,** Efflux of radioactivity through Asc1 by facilitated diffusion in amino acid-free medium. **d,** Efflux of radioactivity through exchange in medium with a saturating concentration of D-serine. **e-f,** Linear conditions to measure efflux by exchange with saturating D-serine in the medium and by diffusion with only DMSO in the medium. Data (mean ± s.e.m.; when not visible error bars are smaller than symbols) from a representative experiment run in triplicates. Consequently, efflux measurements were done in time-periods no longer than 40 s for the exchange mode and at 5 min for the diffusion mode. **g**, Time for efflux quantification in units of time per percentage of the loaded radioactivity (min per % of radioactive load) in HeLa cells transfected with wild-type Asc1 or mutants S246G and Y333F. Data (mean ± s.e.m.) correspond to n=5 (WT facilitated diffusion) or n=3 (rest of efflux measurements) independent experiments. **h**, Cell content (nmol mg^{-1} cell protein) of Asc1-substrate and non-substrate amino acids in HeLa cells co-transfected with wild type, S246G or Y333F Asc1 and CD98hc and incubated for 5 min with 20 µM D-serine. Data (mean ± s.e.m.) from a representative experiment run in triplicates. P-values were obtained by two-tailed Student´s t test: ns, non-significant; (***), $p \leq 0.001$. S246G Gly, p = 0.690; S246G Ala, p = 0.804; S246G Ser, $p = 4.0 \cdot 10^{-4}$; S246G Thr, p = 0.109; S246G total substrate, p = 0.961; S246G total non-substrate, p = 0.677; Y333F Gly, p = 0.338; Y333F Ala, p = 0.541; Y333F Ser, p = 0.820; Y333F Thr, p = 0.254; Y333F total substrate, p = 0.295; Y333F total non-substrate, p = 0.582.

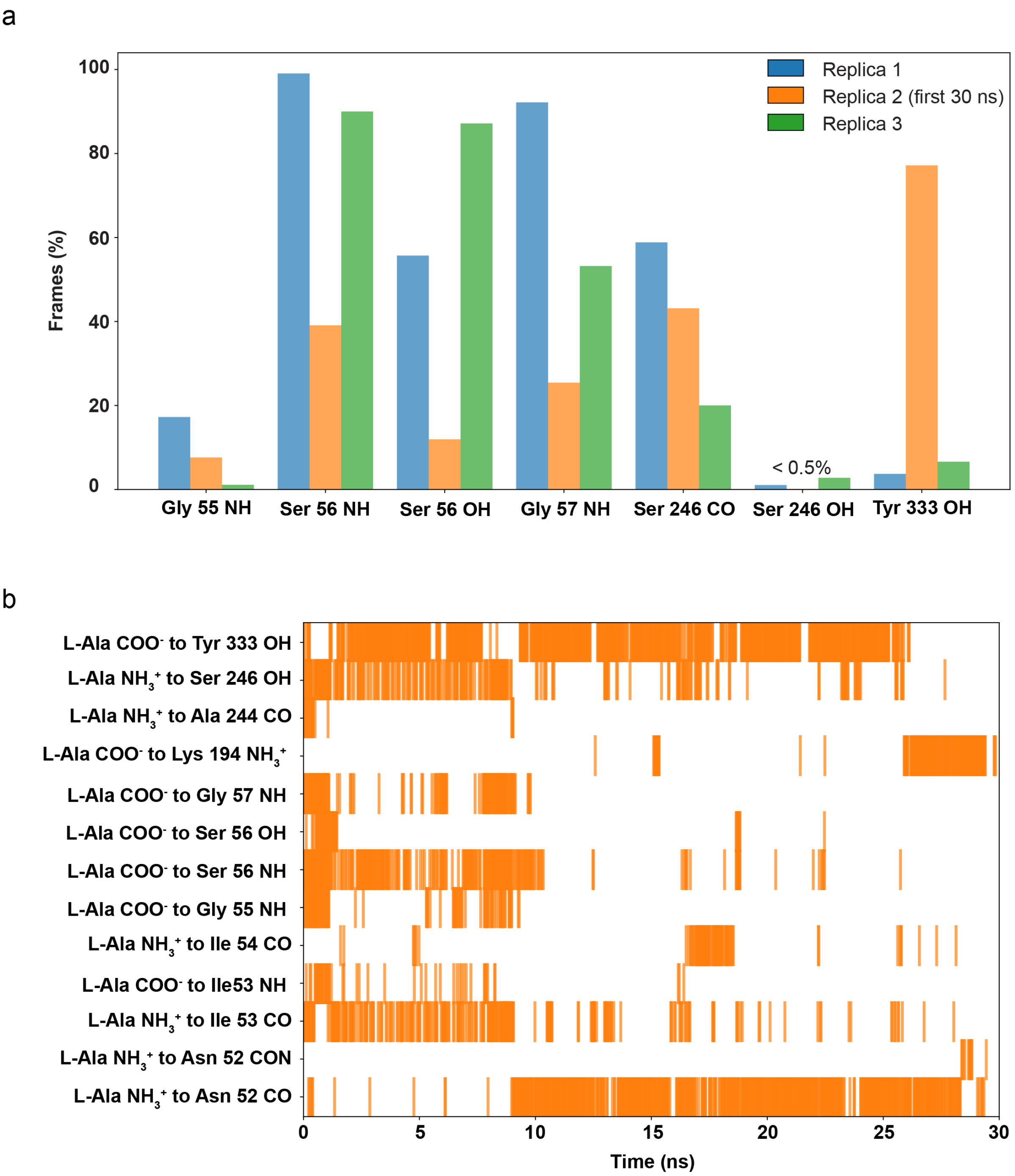

Supplementary Figure 12. **Molecular dynamics analysis of Asc1 apo (PDB ID 8QEY) bound to L-alanine.** Pose 1 for L-alanine in the PELE analysis (**Fig. 3** and **Supplementary Fig. 7**) was the starting structure for the MD analysis. **a**, Percentage of hydrogen bonds of L-alanine with Asc1 residues along the three different replicas of 500 ns (blue, orange and green). **b**, Hydrogen bond presence in the second replica every 0.02 ns, along the first 30 ns of the simulation. In **a** and **b,** the chemical groups interacting with L-alanine are indicated: CO, carbonyl; OH, hydroxyl; NH, amide; CON, carboxamide side chain.

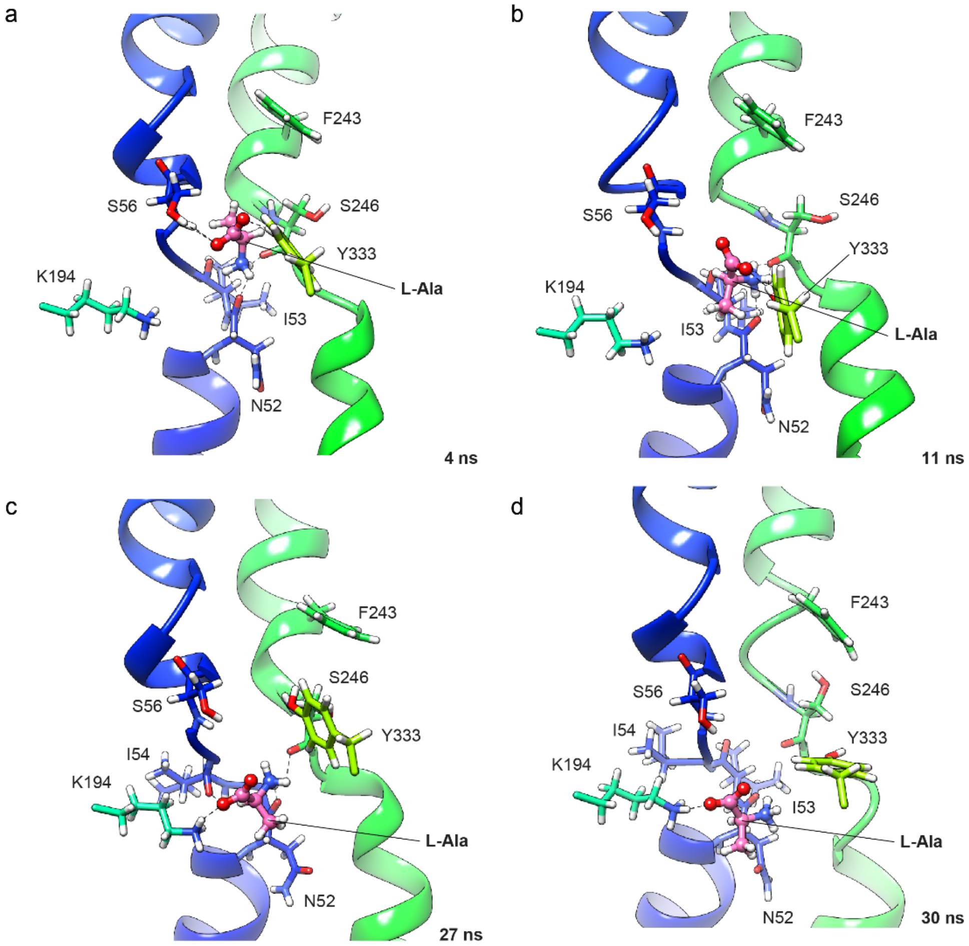

Supplementary Figure 13. **Model for Asc1 substrate release to the cytosol**. Molecular dynamics showed sequential interaction of L-alanine with the indicated residues prior to the release to the cytosol. Snapshots extracted at times **a,** 4 ns, **b,** 11 ns, **c,** 27 ns, and **d,** 30 ns from the orange trajectory (replica 2) shown in Supplementary Fig. 12. Putative H-bonds between L-alanine (magenta) and Asc1 atoms are indicated by a dashed line. C atoms in the Asc1 residues are colored according with the corresponding TM domains as in Fig. 1. All N and O atoms are colored blue and red respectively. The helixes of TM5 (Lys 194) and TM8 (Tyr 333) are omitted for the sake of clarity.

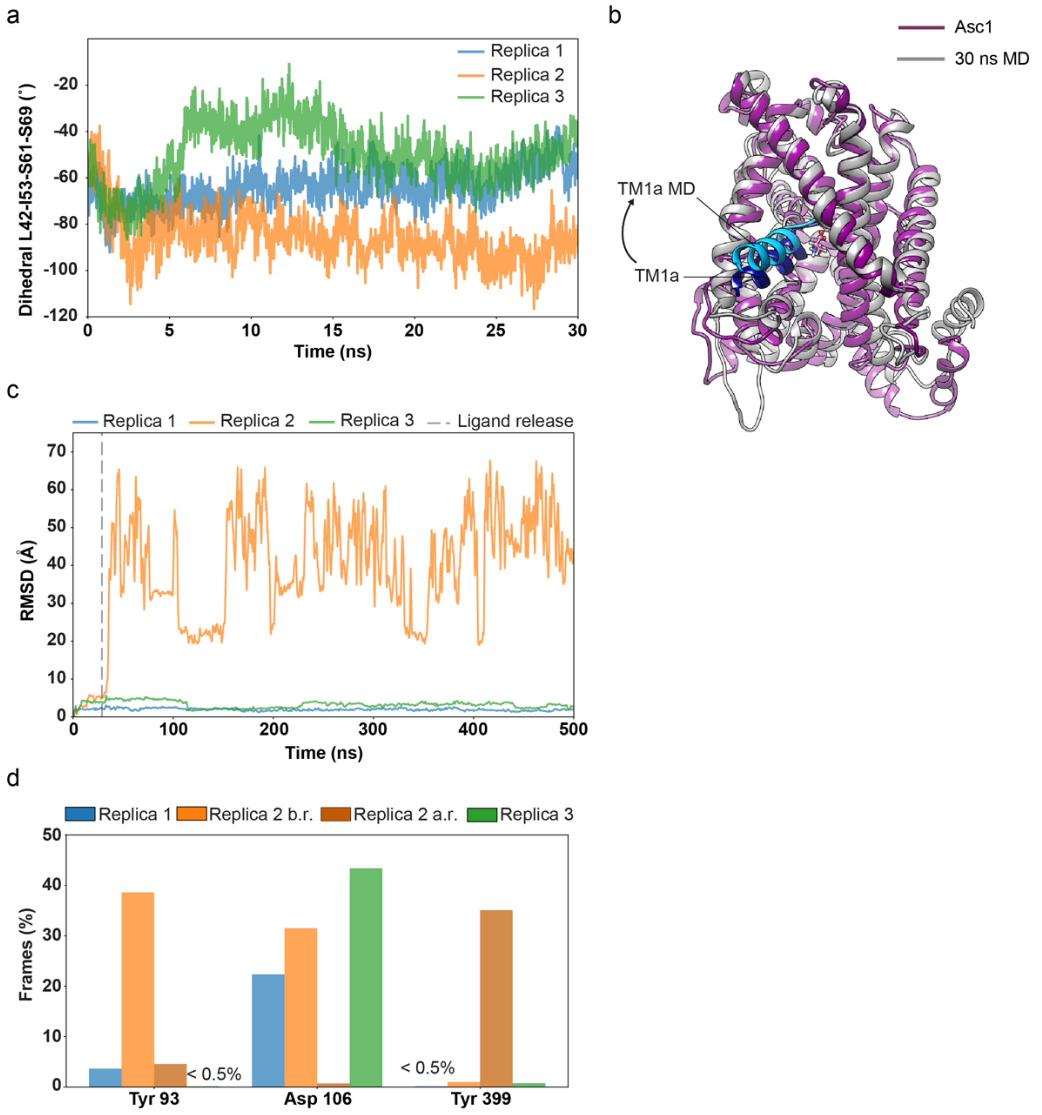

Supplementary Figure 14. Molecular dynamics analysis of Asc1 apo (PDB ID: 8QEY) bound to L-alanine. Pose 1 for L-alanine in the PELE analysis (**Fig. 3** and **Supplementary Figure 7**) was the starting structure for the MD analysis. **a**, Evolution of the angle composed by the axis of TM1a and TM1b (between the α-carbons of the residues indicated in the Y axis) to monitor TM1a conformation along the three replicas (blue, orange and green). **b**, Open conformation of TM1a at 30 ns of the second replica. The experimental structure of Asc1 is colored in dark purple, whereas the 30 ns timepoint of the MD simulation is coloured in grey. TM1a in the experimental structure is highlighted in dark blue, whereas TM1a in the 30 ns timepoint of the MD simulation is highlighted in light blue. **c**, Temporal evolution of the L-alanine substrate rmsd along three different replicas of 500 ns (blue, orange and green). The initial pose in each replica was the reference to calculate rmsd values. The dashed line marks the moment the L-alanine substrate leaves Asc1's cavity (30 ns). For visualization purposes, the data has been smoothed every fifty points of data. **d**, Asn 249 interaction before and after ligand release in the three different replicas of 500 ns. Percentage of hydrogen bonds of Asn 249 with other residues every 0.02 ns. Replica 1 is color-coded in blue and replica 3 in green. The second replica is split between

the first 30 ns before the ligand release (b.r., light orange) and the rest of the simulation after the ligand release (a.r., dark orange). In b and f: i) all the interactions with residues that had < 0.5% of presence were considered residual and not plotted; ii) in the orange replica only the first 30 ns of the trajectory, prior of substrate release is plotted.

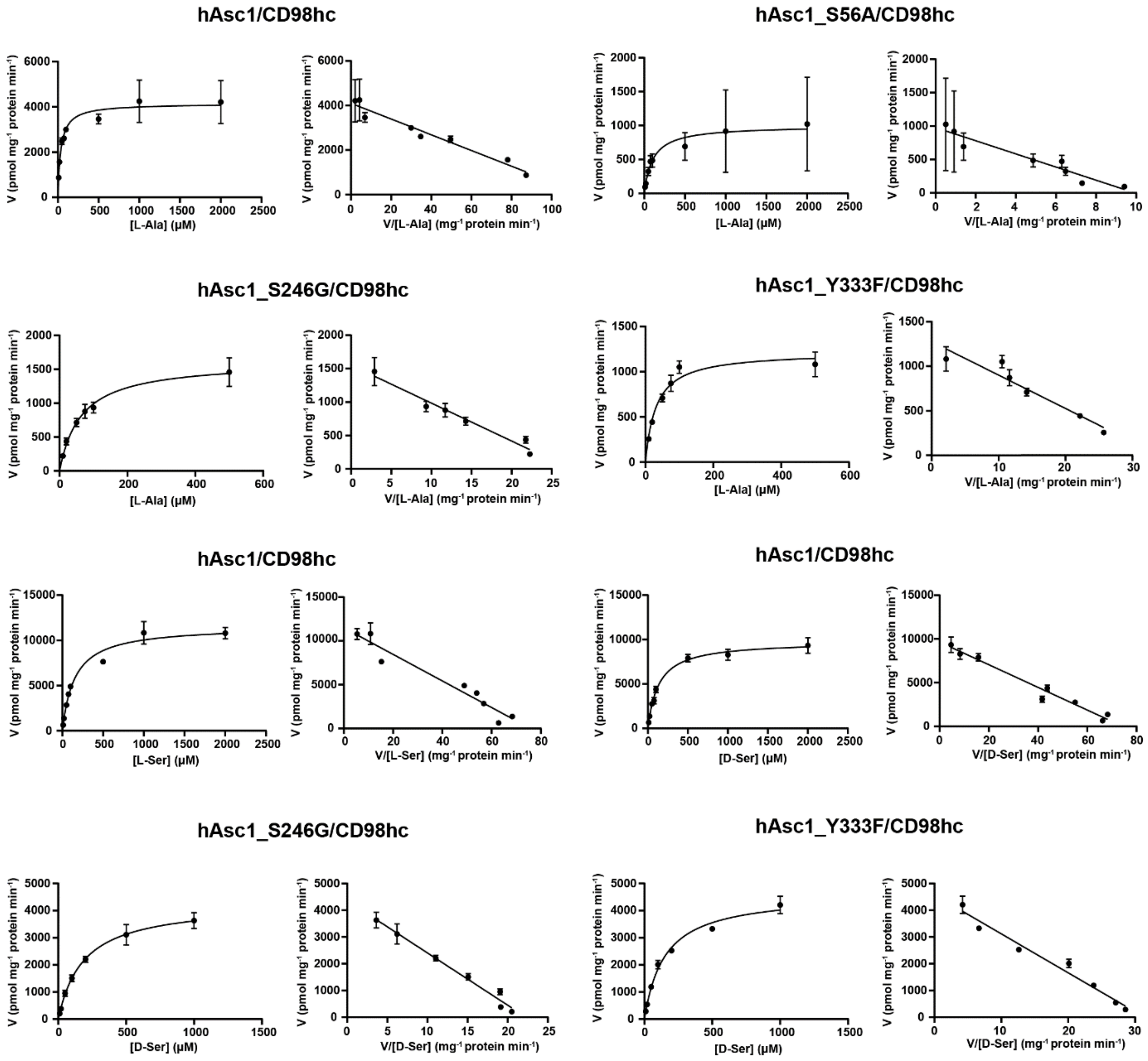

Supplementary Figure 15. Kinetics of the uptake of the indicated amino acids by the indicated hAsc1/CD98hc variants in HeLa cells. Data (mean±SEM) from a representative experiment run in triplicates are shown (left) and Eadie-Hofstee transformation (right).

	human Asc1/CD98hc (EMDB-18379) (PDB 8QEY)
**Data collection and processing**	
Magnification	130,000
Voltage (kV)	300
Electron exposure ($e^{-}Å^{-2}$)	50
Defocus range (μm)	-0.8 ~ -2.4
Pixel size (Å)	0.656
Symmetry imposed	none
Initial particle images (no.)	2,699,978
Final particle images (no.)	139,507
Map resolution (Å)	4.0
FSC threshold	0.143
Map resolution range (Å)	5.1 – 3.7
**Refinement**	
Initial model used (PDB code)	none
Model resolution (Å)	4.1
FSC threshold	0.143
Map sharpening *B* factor ($Å^2$)	-194
Model composition	
Non-hydrogen atoms	7306
Protein residues	918
Ligands	9
B factors ($Å^2$)	
Protein	107.85
Ligand	96.37s
R.m.s. deviations	
Bond lengths (Å)	0.007
Bond angles (°)	1.061
Validation	
MolProbity score	1.85
Clashscore	7.41
Poor rotamers (%)	0.91
Ramachandran plot	
Favored (%)	93.22
Allowed (%)	6.78
Disallowed (%)	0.00

Supplementary Table 1. Cryo-EM data collection, refinement and validation statistics

Uncropped images for Supplementary Figure 1c

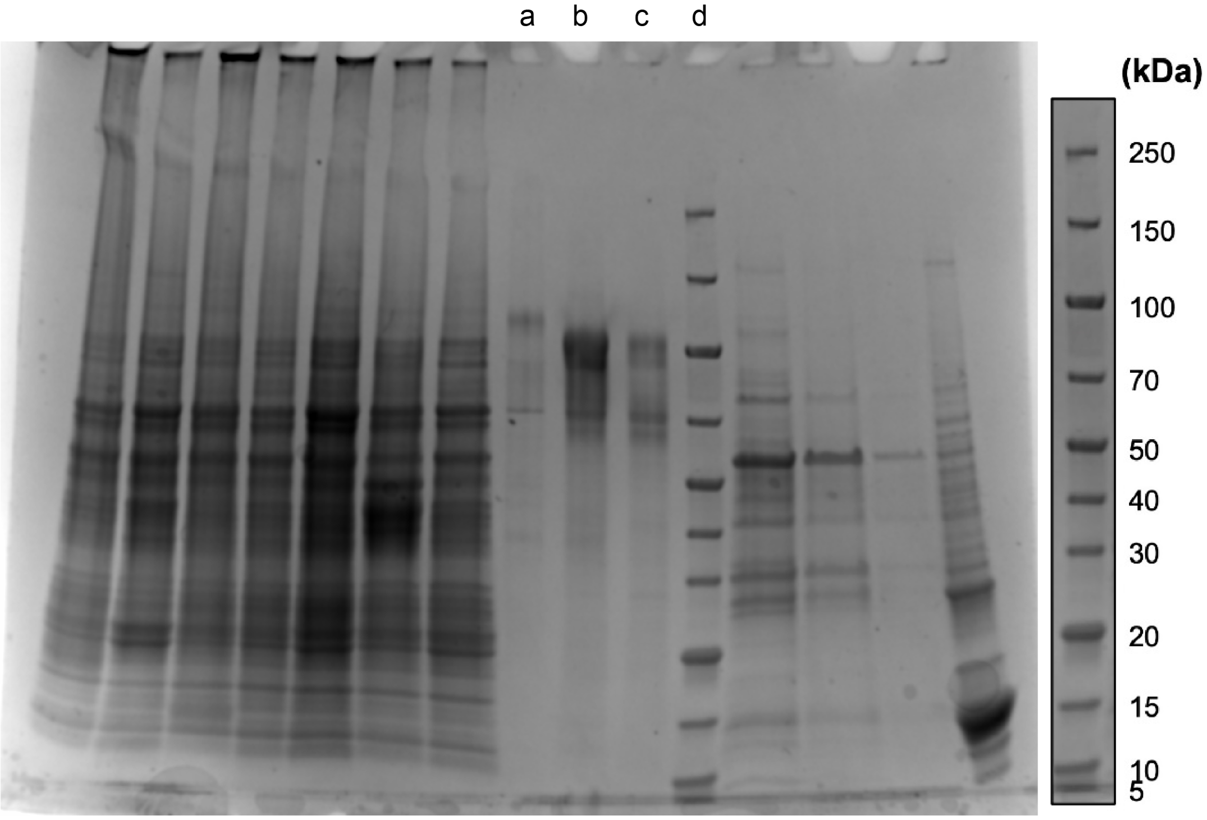

a)-c) Central SEC peak fractions. Fractions b and c are shown in Supplementary Fig. 1c. d) Molecular weight ladder. Other wells: samples unrelated to the project of the paper.

Uncropped images for Supplementary Figure 9b

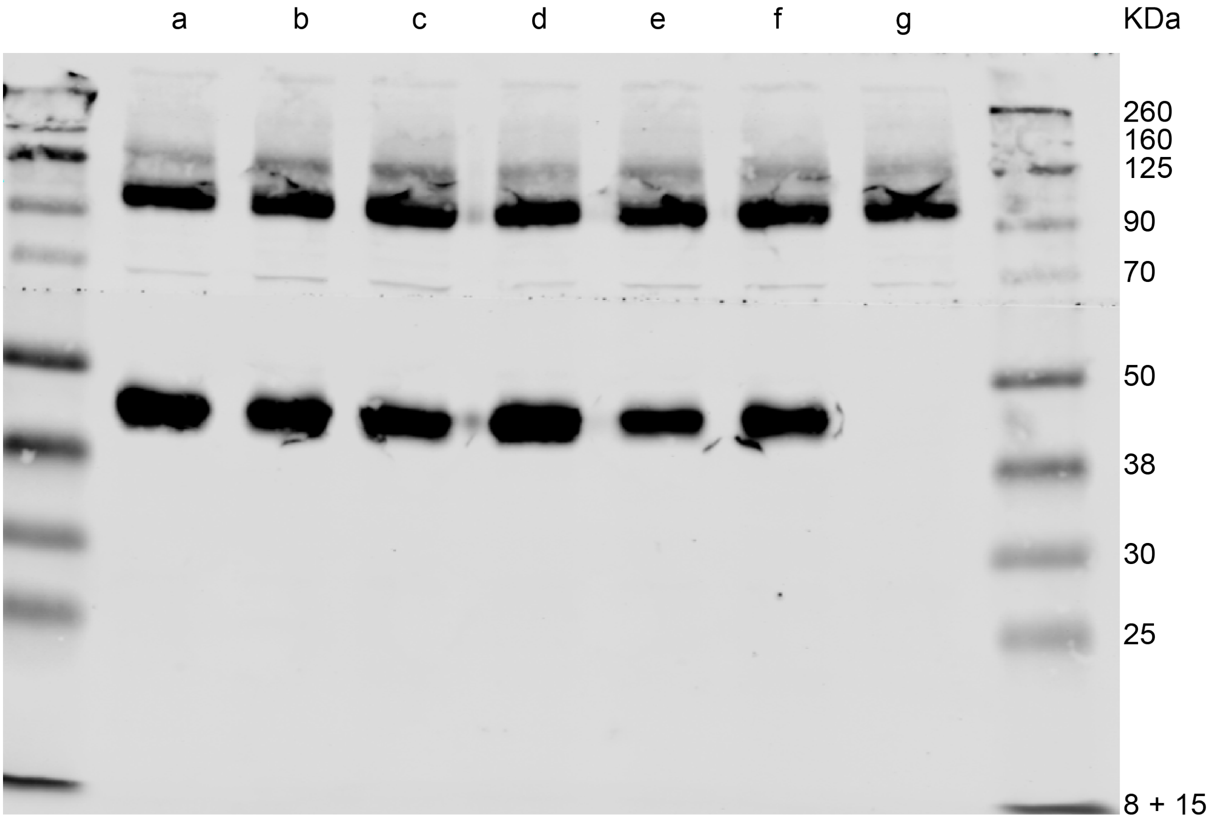

a) Asc1 WT. b) Asc1 S56A. c) Asc1 S246A. d) Asc1 S246G. e) Asc1 Y333S. f) Asc1 Y333F. g) eGFP. Antibodies used: anti-myc tag mouse mAb (05-724, Millipore) 1:1000 dilution in 5% milk, anti-SERCA2 rabbit mAb (D51B11, Cell Signaling Technology®) 1:1000 dilution in 5% milk, goat anti-Mouse IgG (H+L) secondary antibody - DyLight 680 (catalog number 35518, Thermo Fisher Scientific) 1:10000 dilution in 5% milk, goat anti-Mouse IgG (H+L) secondary antibody - DyLight 800 4X PEG (catalog number SA5-35521, Thermo Fisher Scientific) 1:10000 dilution in 5% milk, goat anti-Rabbit IgG (H+L) secondary antibody - DyLight 680 (catalog number 35568, Thermo Fisher Scientific) 1:10000 dilution in 5% milk, goat anti-Rabbit IgG (H+L) secondary antibody - DyLight 800 4X PEG (catalog number SA5-35571, Thermo Fisher Scientific) 1:10000 dilution in 5% milk.

LiCor Odyssey Infrared Imaging System 9120 settings:

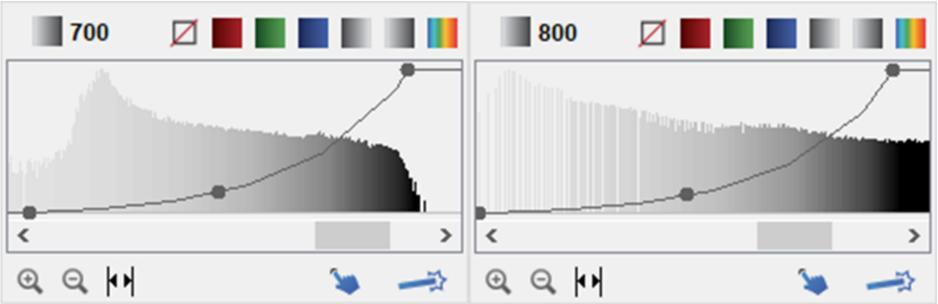

Supplement: Supplementary file 1 — Supplementary Information [file 41467_2024_47385_MOESM1_ESM.pdf]
