# Supplementary material for: Structure and mechanisms of transport of human Asc1/CD98hc amino acid transporter
Source: Nat Commun. 2024 Apr 6;15:2986. doi: 10.1038/s41467-024-47385-3 (PMC10998858; doi:10.1038/s41467-024-47385-3)
Supplement: Supplementary file 3 — Description of Additional Supplementary Files [file 41467_2024_47385_MOESM3_ESM.pdf]

Description of Additional Supplementary Materials

File Name: Supplementary Data 1

Description: Primers used to generate human ASC1 mutants

File Name: Supplementary Data 2

Description: Raw Data and Statistical Source Data for Supplementary Figures
