# Supplementary material for: Structure and mechanisms of transport of human Asc1/CD98hc amino acid transporter
Source: Nat Commun. 2024 Apr 6;15:2986. doi: 10.1038/s41467-024-47385-3 (PMC10998858; doi:10.1038/s41467-024-47385-3)
Supplement: Supplementary file 6 — Reporting Summary [file 41467_2024_47385_MOESM6_ESM.pdf]

Corresponding author(s): Ekaitz Errasti Murugarren, Manuel Palacin, Oscar Llorca

Last updated by author(s): Mar 11, 2024

Reporting Summary

Nature Portfolio wishes to improve the reproducibility of the work that we publish. This form provides structure for consistency and transparency in reporting. For further information on Nature Portfolio policies, see our Editorial Policies and the Editorial Policy Checklist.

Statistics

For all statistical analyses, confirm that the following items are present in the figure legend, table legend, main text, or Methods section.

n/a	Confirmed	
☐	☒	The exact sample size (*n*) for each experimental group/condition, given as a discrete number and unit of measurement
☐	☒	A statement on whether measurements were taken from distinct samples or whether the same sample was measured repeatedly
☐	☒	The statistical test(s) used AND whether they are one- or two-sided<br>*Only common tests should be described solely by name; describe more complex techniques in the Methods section.*
☐	☒	A description of all covariates tested
☐	☒	A description of any assumptions or corrections, such as tests of normality and adjustment for multiple comparisons
☐	☒	A full description of the statistical parameters including central tendency (e.g. means) or other basic estimates (e.g. regression coefficient) AND variation (e.g. standard deviation) or associated estimates of uncertainty (e.g. confidence intervals)
☐	☒	For null hypothesis testing, the test statistic (e.g. *F*, *t*, *r*) with confidence intervals, effect sizes, degrees of freedom and *P* value noted<br>*Give P values as exact values whenever suitable.*
☒	☐	For Bayesian analysis, information on the choice of priors and Markov chain Monte Carlo settings
☒	☐	For hierarchical and complex designs, identification of the appropriate level for tests and full reporting of outcomes
☒	☐	Estimates of effect sizes (e.g. Cohen's *d*, Pearson's *r*), indicating how they were calculated

Our web collection on statistics for biologists contains articles on many of the points above.

Software and code

Policy information about availability of computer code

Data collection	EPU 2.12
Data analysis	R version 4.1.3 , RELION4 , CTFFIND-4.1 , cryoSPARCv3.2.0 , UCSF Chimera v1.16 , UCSF ChimeraX v1.6.1 , coot 0.9.8.5 , LAFTER v1.1 , phenix 1.20-1-4487-000 , Prism v9.0.0, BLAST 2.14.0, Clustal W v1.81, Bioedit v7.7.1, MEGA X v11.0.13, PROPKA 3.0, PrepWizard (Schrödinger Suite 2021-4), CHARMM-GUI Membrane Builder Webserver (version num. N/A), CHARMM36, AMBER v20, Gnuplot v4.6, OPLS2005, Glide (Schrödinger Suite 2021-4), MDtraj V1.9.7, Pymol 2.3.0, CPPTRAJ V4.25.6, PELE V1.7.1

For manuscripts utilizing custom algorithms or software that are central to the research but not yet described in published literature, software must be made available to editors and reviewers. We strongly encourage code deposition in a community repository (e.g. GitHub). See the Nature Portfolio guidelines for submitting code & software for further information.

Data

Policy information about availability of data

All manuscripts must include a data availability statement. This statement should provide the following information, where applicable:

- Accession codes, unique identifiers, or web links for publicly available datasets
- A description of any restrictions on data availability
- For clinical datasets or third party data, please ensure that the statement adheres to our policy

Raw data for all figures and tests is available as supplemental material and source data.

The cryo-EM map has been deposited in the Electron Microscopy Data Bank (EMDB) under the accession code EMD-18379 [https://www.ebi.ac.uk/pdbe/entry/emdb/EMD-18379] (apo human Asc1/CD98hc in inward-facing semi-occluded conformation). The atomic coordinates have been deposited in the Protein Data Bank (PDB) under the accession code 8QEY [https://doi.org/10.2210/pdb8QEY/pdb] (apo human Asc1/CD98hc in inward-facing semi-occluded conformation). The previously-published atomic coordinates referred to in the text, and shown in Figure 2, are available in the Protein Data Bank (PDB) under the accession codes 7B00 [http://doi.org/10.2210/pdb7B00/pdb] (apo human LAT2/CD98hc in inward-facing open conformation) and 7DSQ [http://doi.org/10.2210/pdb7DSQ/pdb] (3,5-diiodo-L-tyrosine-bound human LAT1/CD98hc in outward-facing occluded conformation).
Molecular dynamics trajectories (3 replicas for apo Asc1 and 3 replicas for holo L-alanine bound-Asc1) as well as PELE raw data (initial dockings for L-alanine, L- and D-serine, the final energy minimums shown in the paper, the .conf files needed to run PELE and the run file used to queue the calculations at the MareNostrum supercomputer) have been deposited in a Zenodo.com repository under accession code: [https://doi.org/10.5281/zenodo.10788789].
Primer sequences used for mutagenesis studies are provided in the Supplementary Data 1 file. The source data underlying Figures 3c-4 and Tables 1-2 are provided as a Source Data File; and Supplementary Figures 1b, 8e, 8f, 9c, 11e, 11f, 11g, 11h and 15 are provided in the Supplementary Data 2 file. All other data generated in this study is available within the Supplementary Information file.

Research involving human participants, their data, or biological material

Policy information about studies with human participants or human data. See also policy information about sex, gender (identity/presentation), and sexual orientation and race, ethnicity and racism.

Reporting on sex and gender	N/A
Reporting on race, ethnicity, or other socially relevant groupings	N/A
Population characteristics	N/A
Recruitment	N/A
Ethics oversight	N/A

Note that full information on the approval of the study protocol must also be provided in the manuscript.

Field-specific reporting

Please select the one below that is the best fit for your research. If you are not sure, read the appropriate sections before making your selection.

☒ Life sciences ☐ Behavioural & social sciences ☐ Ecological, evolutionary & environmental sciences

For a reference copy of the document with all sections, see nature.com/documents/nr-reporting-summary-flat.pdf

Life sciences study design

All studies must disclose on these points even when the disclosure is negative.

Sample size	At least three biological independent replicates were obtained for each condition in every experiment, as stated in the Methods section and figure legends. Due to the reproducibility of the results, with a clear effect observed and low variability, three independent experiments were deemed enough for the functional studies. For the cryo-EM structural data, data set size was determined based on the microscope availability. Thus, collected data was sufficient to obtained maps with the reported resolution and performed the model building.
Data exclusions	No functional data were excluded from the studies. For the cryo-EM structural data, poor quality micrographs and bad particles were removed during the data processing following standard analysis.
Replication	Reproducibility was assessed through the corresponding statistical test, for the functional experiments. All functional assays were replicated at least three times in independent experiments. All attempts at replication were successful. For the structural studies, quality of the purified protein sample was reproducible in the independent purification replicates.
Randomization	No randomization was needed. As stated in the Methods section, for functional uptake assays normalised to WT glycine or L-serine transport, the experimental batch was included in the model as a covariate. For the rest of the analyses no covariate existed.
Blinding	No blinding was needed in any experiment given that there is no influence of the researcher upon the measurements.

Reporting for specific materials, systems and methods

We require information from authors about some types of materials, experimental systems and methods used in many studies. Here, indicate whether each material, system or method listed is relevant to your study. If you are not sure if a list item applies to your research, read the appropriate section before selecting a response.

Materials & experimental systems

n/a	Involved in the study	
☐	☒	Antibodies
☐	☒	Eukaryotic cell lines
☒	☐	Palaeontology and archaeology
☒	☐	Animals and other organisms
☒	☐	Clinical data
☒	☐	Dual use research of concern
☒	☐	Plants

Methods

n/a	Involved in the study	
☒	☐	ChIP-seq
☒	☐	Flow cytometry
☒	☐	MRI-based neuroimaging

Antibodies

Antibodies used

Anti-Myc-tag mouse monoclonal antibody (catalog number 05-724, clone 4A6, Millipore), anti-SERCA2 rabbit monoclonal antibody (catalog number 9580, clone D51B11, Cell Signaling Technology®), goat anti-Mouse IgG (H+L) Secondary Antibody - DyLight 680 (catalog number 35518, Thermo Fisher Scientific), goat anti-Mouse IgG (H+L) Secondary Antibody - DyLight 800 4X PEG (catalog number SA5-35521, Thermo Fisher Scientific), goat anti-Rabbit IgG (H+L) Secondary Antibody - DyLight 680 (catalog number 35568, Thermo Fisher Scientific), goat anti-Rabbit IgG (H+L) Secondary Antibody - DyLight 800 4X PEG (catalog number SA5-35571, Thermo Fisher Scientific).

Validation

Anti-Myc-tag mouse monoclonal antibody (05-724, Millipore) was validated by Millipore by Western Blot. 0.5-2 µg/mL of this antibody detected Myc-tagged recombinant protein in sequence contexts not well recognized by anti-Myc Tag, clone 9E10 (Catalog # 05-419). Lysates from transfected NIH/3T3 cells were resolved by electrophoresis, transferred to PVDF and probed with anti-Myc Tag, clone 4A6 (1.0ug/mL) Proteins were visualized using a goat anti-mouse secondary conjugated to HRP and chemiluscence detection system. Anti-SERCA2 rabbit monoclonal antibody (D51B11, Cell Signaling Technology®) was validated by Cell Signaling Technology by Western Blot. This antibody detected SERCA2 in cell extracts of RD, NIH/3T3 and C2C12 cell lines.

Eukaryotic cell lines

Policy information about cell lines and Sex and Gender in Research

Cell line source(s)

Two cell lines were used: HeLa (catalog number CCL-2, ATCC®), immortalized cervical carcinoma cells originating from a sample from Henrietta Lacks taken in 1951; and HEK293-6E (CVCL_HF20, licensed through the National Research Council of Canada), immortalized embryonic kidney cells first engineered in 1973, with further modification in 2008 to constitutively express truncated EBNA-1 to improve transient transfection efficiency.

Authentication

The cell lines used were commercial and thus were not authenticated.

Mycoplasma contamination

All cell lines tested negative for mycoplasma contamination throughout the studies.

Commonly misidentified lines
(See ICLAC register)

No commonly misidentified cell lines were used in this study.

Plants

Seed stocks

Report on the source of all seed stocks or other plant material used. If applicable, state the seed stock centre and catalogue number. If plant specimens were collected from the field, describe the collection location, date and sampling procedures.

Novel plant genotypes

Describe the methods by which all novel plant genotypes were produced. This includes those generated by transgenic approaches, gene editing, chemical/radiation-based mutagenesis and hybridization. For transgenic lines, describe the transformation method, the number of independent lines analyzed and the generation upon which experiments were performed. For gene-edited lines, describe the editor used, the endogenous sequence targeted for editing, the targeting guide RNA sequence (if applicable) and how the editor was applied.

Authentication

Describe any authentication procedures for each seed stock used or novel genotype generated. Describe any experiments used to assess the effect of a mutation and, where applicable, how potential secondary effects (e.g. second site T-DNA insertions, mosiacism, off-target gene editing) were examined.
