# Supplementary material for: Structure and mechanisms of transport of human Asc1/CD98hc amino acid transporter
Source: Nat Commun. 2024 Apr 6;15:2986. doi: 10.1038/s41467-024-47385-3 (PMC10998858; doi:10.1038/s41467-024-47385-3)
Supplement: Supplementary file 2 — Peer Review File [file 41467_2024_47385_MOESM2_ESM.pdf]

Peer Review File

Structure and mechanisms of transport of human Asc1/CD98hc amino acid transporter

Open Access This file is licensed under a Creative Commons Attribution 4.0 International License, which permits use, sharing, adaptation, distribution and reproduction in any medium or format, as long as you give appropriate credit to the original author(s) and the source, provide a link to the Creative Commons license, and indicate if changes were made. In the cases where the authors are anonymous, such as is the case for the reports of anonymous peer reviewers, author attribution should be to 'Anonymous Referee' followed by a clear attribution to the source work. The images or other third party material in this file are included in the article's Creative Commons license, unless indicated otherwise in a credit line to the material. If material is not included in the article's Creative Commons license and your intended use is not permitted by statutory regulation or exceeds the permitted use, you will need to obtain permission directly from the copyright holder. To view a copy of this license, visit http://creativecommons.org/licenses/by/4.0/.

Reviewer #1 (Remarks to the Author):

Rullo-Tubau et al. report the cryo-EM structure of the Asc1-CD98hc complex (also known as Asc1-4F2hc or by the solute carrier nomenclature, SLC7A10-SLC3A2). This transporter complex can operate both through exchange (which is typical of the heteromeric amino acid transporters HAT) and outward-oriented facilitated transport (which is unique to Asc1).
They determine a cryoEM structure at about 3.6 Å resolution for the apoform solubilized in digitonin (substrates even at 10mM did not show binding). Furthermore, they analyse and compare to alphafold-derived structure and assess ligand binding and conformational analysis through “Protein Energy Landscape Exploration” (PELE) analysis. They also do thorough functional studies using cellular uptake and extrusion assays. They find in particular Tyr333 and Ser246 to play pivotal roles in the transport mechanism, both in the exchange and facilitated diffusion mode.This is an exciting study of an important transporter, and the combination of experimental and computational studies and a careful use of the literature reach important, new insights and discussion. The manuscript is a bit long in places and could help from a revision to impose a more concise style
The following are suggestions to improve the manuscript.

Major points:
1. It is of concern that no substrate binding can be detected in the cryo-EM structures. How does that fit substrate binding properties outside of the cryo-EM conditions? Will specific lipids, cholesterol or detergents be better suited, and might other sample presentation modes or substrate complexes be better suited for amino acid substrate binding to be observed? I would like the authors to comment on this in the manuscript
2. Also, knowing that the structure represents a form that does not bind substrate, some disclaimers must be included in the structural analysis and PELE analysis. For the same reason, the more speculative parts of substrate binding and conformational changes associated with transport can be shortened
3. Specific mentions of hydrogen bonds should be avoided given that only a moderate resolution has been ontained - or at least the description should be modified, e.g. stating “possible hydrogen bond between X and Y” etc.

Reviewer #2 (Remarks to the Author):

The paper by Rullo and Martinez describes the cryo-EM structure of human Asc1/ CD98hc complex. The structure of the Asc1/CD98hc heterodimer in the apo-state was analyzed by cryo-EM at ~4 Å resolution. The authors incubated the purified proteins with a high substrate concentration, D-Seri, but could not observe a cryo-EM density representing the substrate. Hence, the structure represents the apo-state with no substrate bound. The authors found that the Asc1/CD98hc structure, with the CD98hc ectodomain atop Asc1's transmembrane helices, is covalently linked via a disulfide bridge. They also observed four N-glycosylation sites on CD98hc. This structure contrasts with other HAT structures, especially in the positioning of TM1a, TM5, and TM7, resulting in a partially occluded Asc1/CD98hc conformation. The structure's comparison with AlphaFold predictions highlighted discrepancies in key functional regions predicted by the authors. In an inward-facing, partially occluded conformation, the substrate binding site is formed by TMs 1, 1a, 6, 6b, and 8, with notable contributions from a 55G(S/T)G57 motif and Ser 246. Tyr 333 in TM8, unique to Asc1, is crucial for substrate selectivity and translocation. Asc1, exclusive to vertebrates and featuring the conserved Tyr 333, is distinct in substrate binding and transport functions compared to other vertebrate LATs. Molecular docking and PELE studies predicted minimal binding-energy modes for amino acids, revealing key interactions in the substrate recognition process. These studies indicated that Asc1 binds substrates between TM1 and TM6's unwound regions, with key residues in TM1, TM6, and TM8 involved in substrate recognition. Functional studies of Asc1 mutants (S56A, S246A, S246G, Y333S, Y333F) showed dramatic decreases in small neutral substrate uptake in HeLa cells, emphasizing the

importance of Ser 56, Ser 246, and Tyr 333 in transporting small neutral amino acids. Asc1/CD98hc operates in amino acid exchange and facilitated diffusion modes, with Tyr 333 possibly playing a role in the latter. Efflux experiments with D-serine highlighted the importance of Ser 246 and Tyr 333 in both transport modes. Mutations in these residues resulted in longer transitions from outward- to inward-facing conformations, suggesting their role in transporter occlusion. Molecular dynamics simulations with L-alanine-bound Asc1 showed the substrate remained bound between TM1 and TM6, with Tyr 333 playing a variable role in substrate interactions. The substrate release mechanism involved the opening of the cytosolic vestibule, facilitated by the substrate's disconnection from TM1's unwound region. Finally, the kinetic analysis of Asc1 mutants revealed impacts on the kinetic parameters of L-alanine and D-serine transport, with mutations affecting binding energy and translocation efficiency. The proposed mechanistic model suggests Ser 56's role in fixing the substrate in a productive pose, while Tyr 333's rotation towards TM6 facilitates substrate movement. This model supports the specific roles of these residues in substrate translocation within Asc1. The mechanistic understanding would have been much deeper had the authors succeeded in obtaining the structures in complex with substrates. Nevertheless, the authors predict the substrate binding modes by docking and simulations.

-What could be the functional role of CD98hc other than trafficking, given that there are some interactions?
-What could be the reason this transporter did not capture any substrate despite incubating the purified proteins with an excessive amount of D-Ser? Could detergent micelle interfere with the substrate uptake into the core of the Asc-1 proteins in the authors' sample preparation? Alternatively, is the turnover rate so fast that the occluded state is hard to capture?
-Can the MD simulations predict substrate-specific stabilization of functional states? It is hard to understand what underlies the mechanism of stereo-specificity (e.g., D-Ser vs. L-Ser).
-Traynelis review (#14) is outdated. Hansen et al. (Pharmacol Rev. 2021 Oct;73(4):298-487. doi: 10.1124/pharmrev.120.000131.) should be cited instead.

Point-by-point response to reviewers

Reviewer #1

Rullo-Tubau et al. report the cryo-EM structure of the Asc1-CD98hc complex (also known as Asc1-4F2hc or by the solute carrier nomenclature, SLC7A10-SLC3A2). This transporter complex can operate both through exchange (which is typical of the heteromeric amino acid transporters HAT) and outward-oriented facilitated transport (which is unique to Asc1).
They determine a cryoEM structure at about 3.6 Å resolution for the apoform solubilized in digitonin (substrates even at 10mM did not show binding). Furthermore, they analyse and compare to alphafold-derived structure and assess ligand binding and conformational analysis through "Protein Energy Landscape Exploration" (PELE) analysis. They also do thorough functional studies using cellular uptake and extrusion assays. They find in particular Tyr333 and Ser246 to play pivotal roles in the transport mechanism, both in the exchange and facilitated diffusion mode.This is an exciting study of an important transporter, and the combination of experimental and computational studies and a careful use of the literature reach important, new insights and discussion. The manuscript is a bit long in places and could help from a revision to impose a more concise style
The following are suggestions to improve the manuscript.

We thank the reviewer for her/his positive comments and suggestions, which have helped us to improve the manuscript.

Major points:
1. It is of concern that no substrate binding can be detected in the cryo-EM structures. How does that fit substrate binding properties outside of the cryo-EM conditions? Will specific lipids, cholesterol or detergents be better suited, and might other sample presentation modes or substrate complexes be better suited for amino acid substrate binding to be observed? I would like the authors to comment on this in the manuscript

We appreciate the suggestion to clarify this issue in the manuscript. We have modified the text accordingly adding our thoughts on the matter in the discussion section.

There might be two reasons for why we have obtained an apo structure: due to the narrowing of the cytosolic vestibule and/or the intrinsic low affinity of the inward facing conformation of LATs and HATs. Apparent affinities for Asc1/CD98hc have been measured in oocytes (*Y. Fukusawa et al, J. Biol. Chem., 2000*) and HeLa cells (the present work) and are in the micromolar range. However, these are exofacial K_ms. A characteristic of HATs is the asymmetry in substrate interaction that allows them to harmonize intra- and extracellular pools, hence having micromolar exofacial apparent affinities and millimolar endofacial apparent affinities (*E. Errasti-Murugarren et al, Nat. Commun., 2019*; *G. Gauthier-Coles et al, Nat. Commun., 2021*). Thus, the lack of

substrate binding in our structure would be consistent with 10 mM of D-serine not being enough to saturate the low-affinity inward-facing conformation.

We have reasons to hypothesise that detergent, particularly steroid-like detergents such as digitonin and glycol-diosgenin, stabilise these low-affinity inward-facing conformations (see the answer to comment #2 of reviewer #2 for an in-depth review of existing proof that could support this hypothesis). Hence, we believe that strategies like the one recently developed for LAT1/CD98hc (*Y. Lee et al, bioRxiv 2023.12.03.567112*), which was reconstituted into nanodisc-encased lipids and allowed the solving of outward-facing and occluded conformations with good ligand density, could also prove useful for Asc1/CD98hc. Notably, this study also found particles of inward-facing LAT1/CD98hc, which were in an apo-state that the authors of the manuscript attributed to the low affinity of this conformation for substrate.

2. Also, knowing that the structure represents a form that does not bind substrate, some disclaimers must be included in the structural analysis and PELE analysis. For the same reason, the more speculative parts of substrate binding and conformational changes associated with transport can be shortened

We agree with the proposed changes by the reviewer and have accordingly added disclaimers in the Results and Discussion sections as well as shortening the more speculative descriptions in the text (changes are highlighted in a different color in the revised version).

Nevertheless, we believe that the lack of substrate in our structure does not preclude it from being used to analyse ligand binding through PELE analysis. The PELE experiments start by docking the substrate inside the substrate binding cavity and the substrate and the structure is then allowed to move, thus overcoming the limitations of having determined a semi-occluded structure where the substrate could not reach the substrate binding site.

Moreover, possible interacting residues identified by PELE were confirmed by functional assays to be of functional importance for Asc1, suggesting that the use of the structure we determined as initial point for the PELE analysis allow to predict how substrates are recognised by Asc1.

3. Specific mentions of hydrogen bonds should be avoided given that only a moderate resolution has been obtained - or at least the description should be modified, e.g. stating "possible hydrogen bond between X and Y" etc.

Done. We have modified the text to have language that is more careful when speaking about the putative bonds predicted in our structure.

Reviewer #2

The paper by Rullo and Martinez describes the cryo-EM structure of human Asc1/ CD98hc complex. The structure of the Asc1/CD98hc heterodimer in the apo-state was analyzed by cryo-EM at ~4 Å resolution. The authors incubated the purified proteins with a high substrate concentration, D-Seri, but could not observe a cryo-EM density representing the substrate. Hence, the structure represents the apo-state with no

substrate bound. The authors found that the Asc1/CD98hc structure, with the CD98hc ectodomain atop Asc1's transmembrane helices, is covalently linked via a disulfide bridge. They also observed four N-glycosylation sites on CD98hc. This structure contrasts with other HAT structures, especially in the positioning of TM1a, TM5, and TM7, resulting in a partially occluded Asc1/CD98hc conformation. The structure's comparison with AlphaFold predictions highlighted discrepancies in key functional regions predicted by the authors. In an inward-facing, partially occluded conformation, the substrate binding site is formed by TMs 1, 1a, 6, 6b, and 8, with notable contributions from a 55G(S/T)G57 motif and Ser 246. Tyr 333 in TM8, unique to Asc1, is crucial for substrate selectivity and translocation. Asc1, exclusive to vertebrates and featuring the conserved Tyr 333, is distinct in substrate binding and transport functions compared to other vertebrate LATs. Molecular docking and PELE studies predicted minimal binding-energy modes for amino acids, revealing key interactions in the substrate recognition process. These studies indicated that Asc1 binds substrates between TM1 and TM6's unwound regions, with key residues in TM1, TM6, and TM8 involved in substrate recognition. Functional studies of Asc1 mutants (S56A, S246A, S246G, Y333S, Y333F) showed dramatic decreases in small neutral substrate uptake in HeLa cells, emphasizing the importance of Ser 56, Ser 246, and Tyr 333 in transporting small neutral amino acids. Asc1/CD98hc operates in amino acid exchange and facilitated diffusion modes, with Tyr 333 possibly playing a role in the latter. Efflux experiments with D-serine highlighted the importance of Ser 246 and Tyr 333 in both transport modes. Mutations in these residues resulted in longer transitions from outward- to inward-facing conformations, suggesting their role in transporter occlusion. Molecular dynamics simulations with L-alanine-bound Asc1 showed the substrate remained bound between TM1 and TM6, with Tyr 333 playing a variable role in substrate interactions. The substrate release mechanism involved the opening of the cytosolic vestibule, facilitated by the substrate's disconnection from TM1's unwound region. Finally, the kinetic analysis of Asc1 mutants revealed impacts on the kinetic parameters of L-alanine and D-serine transport, with mutations affecting binding energy and translocation efficiency. The proposed mechanistic model suggests Ser 56's role in fixing the substrate in a productive pose, while Tyr 333's rotation towards TM6 facilitates substrate movement. This model supports the specific roles of these residues in substrate translocation within Asc1. The mechanistic understanding would have been much deeper had the authors succeeded in obtaining the structures in complex with substrates. Nevertheless, the authors predict the substrate binding modes by docking and simulations.

We thank the reviewer for her/his positive comments and suggestions, which have helped us to improve the manuscript.

-What could be the functional role of CD98hc other than trafficking, given that there are some interactions?

This is a very relevant question. To the best of our knowledge, proper trafficking and localisation of the heterodimer is the only solidly stablished functional role of CD98hc. Previous studies have shown that the light subunit of LAT2 (SLC7A8) cannot be purified with detergent or reconstituted into proteoliposomes unless it is co-expressed with CD98hc, suggesting that interaction with the heavy subunit is essential for stability in a non-native environment. (*A. Rosell et al, Proc. Natl. Acad. Sci. U.S.A., 2014*). The same study found that expression of LAT2 alone in *P. Pastoris* is sufficient to drive uptake of L-

leucine to levels comparable to those of LAT2-CD98hc co-expression, and kinetic analysis revealed similar apparent affinity values for both situations. Together these results indicate that LAT2 alone can be properly folded and trafficked in yeast and that interaction with the heavy chain does not influence LAT2 substrate affinity in a cellular context. In this line, similar results were obtained in a study that showed that LAT1 (SLC7A5) could not be reconstituted in a functional manner in proteoliposomes in the absence of CD98hc (*R. Yan et al, Nature, 2019*).

Nevertheless, other studies have been able to reconstitute LAT1 alone in proteoliposomes and have demonstrated that this monomer is functional (*L. Napolitano et al, Int. J. Biochem. Cell Biol., 2015*; and *L. Napolitano et al, Biochim. Biophys. Acta Gen. Subj., 2017*). Further studies of LAT1 and LAT2 transfected in *P. Pastoris* have shown that, in contrast with previous data, association with CD98hc impacts both the apparent affinity and specificity of these transporters (*S. Kantipudi et al, Int. J. Mol. Sci., 2020*).

Finally, a role of CD98hc in the biogenesis of the heterodimer can be imagined, although to date there are no studies on this subject. The other HAT heavy chain rBAT (SLC3A1) does in fact have this function, and it has been shown that rBAT degrades in the endoplasmic reticulum unless a heterodimer is formed with a light chain (in the case of the study, B^{0+}AT [SLC7A9]) (*P. Bartoccioni et al, Hum. Mol. Genet., 2008*).

Unfortunately, there is not a clear understanding of the mechanisms underlying the role of CD98hc in the transport kinetics of the associated transporters. Indeed, none of the papers describing the atomic structure of HATs, including our present manuscript, reveals structural elements that have an impact in the transport properties. This is why we have not commented in the manuscript on the role of CD98hc in the transport activity of their associated transporters including Asc1.

-What could be the reason this transporter did not capture any substrate despite incubating the purified proteins with an excessive amount of D-Ser? Could detergent micelle interfere with the substrate uptake into the core of the Asc-1 proteins in the authors' sample preparation? Alternatively, is the turnover rate so fast that the occluded state is hard to capture?

As for reviewer 1, we thank for this comment. We hypothesise two reasons for the lack of substrate in our structure: the narrowing of the cytosolic vestibule in the semi-occluded conformation and the intrinsic low substrate affinity of inward-facing conformations. Ever since cryo-EM structures of HATs started to be published we have hypothesised that the detergent used to purify the proteins could be stabilising inward-facing open conformations, perhaps explaining why the first structures of LAT1/CD98hc, LAT2/CD98hc, xCT/CD98hc and B^{0+}AT/rBAT were all obtained in inward-open conformations. Supporting this idea is the fact that steroid-like detergents such as digitonin (Y. Lee et al, Nat. Struct. Mol. Biol., 2019; D. Wu et al, Proc. Natl. Acad. Sci. U.S.A., 2020; C. F. Rodríguez et al, Proc. Natl. Acad. Sci. U.S.A., 2021) and glyco-diosgenin (R. Yan et al, Nature, 2019; R. Yan et al, Sci. Adv., 2020; R. Yan et al, Cell Discov., 2020) were used to purify most of these transporters, and in some cases detergent molecules were found to bind to a known cholesterol binding site between TM10 and TM12 of the light subunit. Moreover, cholesterol has been reported to be necessary for the stability of LAT1/CD98hc purified in detergent micelles (D. Dickens et al, Sci. Rep., 2017).

A particular characteristic of HATs is the asymmetry in substrate interaction, with micromolar apparent affinities in the extracellular face of the transporter and millimolar apparent affinities in the intracellular side, thus enabling them to act as harmonisers that equilibrate the extra- and intracellular amino acid pools (E. Errasti-Murugarren et al, Nat. Commun., 2019; G. Gauthier-Coles et al, Nat. Commun., 2021). Stabilisation of inward-facing structures would mean that the millimolar concentrations of substrate used in cryo-EM preparations, though sufficient to saturate the outward-facing state, would be insufficient to saturate the transporter, perhaps explaining why these HAT structures often have weak density for the ligand. Supporting this hypothesis, a recent study has purified LAT1/CD98hc in a lipid-nanodisc environment instead of detergent, and has managed to obtain outward-facing conformations with robust density for various ligands (Y. Lee et al, bioRxiv 2023.12.03.567112). Additionally, they have observed that in these novel outward-facing conformations, the change in positioning of TM10 and TM12 breaks up the cholesterol binding site, providing stimulating clues that cholesterol and steroid detergents might be stabilising specifically the inward-facing conformation.

Turnover rate could indeed be a factor hampering efforts to obtain occluded conformations. In this line, to date the only native occluded conformation (i.e. not deformed by interaction with inhibitors) has been obtained with LAT1/CD98hc in a nanodisc environment with the poor substrate 2-amino-2-norbornanecarboxylic acid (BCH) (Y. Lee et al, bioRxiv 2023.12.03.567112). The slower kinetics of BCH transport probably helped capture the otherwise transient occluded state of the transporter.

As indicated in the answers to reviewer 1, we have added a comment at the end of the Discussion section (labelled with a different colour in the revised version), suggesting that cryo-EM structures in lipids (i.e., nanodiscs) might allow the transporters to explore difference conformational states as has been recently indicated for human LAT1/CD98hc (Y. Lee et al, bioRxiv 2023.12.03.567112).

-Can the MD simulations predict substrate-specific stabilization of functional states? It is hard to understand what underlies the mechanism of stereo-specificity (e.g., D-Ser vs. L-Ser).

This is an important question given the role of Asc1 in transporting the NMDA receptor coagonist D-serine. The fact is that Asc1 does not present stereoselectivity for L- versus D-serine. Moreover, PELE analysis identified very similar interaction with residues in Asc1 for both substrates. Interestingly, functional analysis of selected mutants of these residues has an identical or similar inactivating effect in the transport of L- and D-serine. Thus, the design of the binding site has been sculpted by evolution to transport small neutral amino acids in a non-stereoselective manner. To clarify this issue, we have changed the previous sentences "*All three residues [S56, S246 and Y333] are nevertheless also essential in the transport of L-alanine and even non-chiral glycine. These observations may indicate that the drastic effect of these mutations on the basic function of the transporter masks any differential effects they could have on stereoselectivity*" (lines 264-267) for "*We hypothesize that evolution has sculpted the binding site of Asc1 to transport small neutral amino acids in a non-stereoselective manner, with three essential interactors that are shared between serine enantiomers*" (labelled with a different colour in the revised version).

Substrate-induced conformational changes would be hard to predict through MD. These simulations should couple conformational shift with entry and/or release of substrate to/from the binding site and would be computationally very intensive. In light of our

experimental results, in which mutation of binding site residues do not alter the stereoselectivity of the transporter, we are not sure that simulations would help clarify this mechanism.

-Traynelis review (#14) is outdated. Hansen et al. (Pharmacol Rev. 2021 Oct;73(4):298-487. doi: 10.1124/pharmrev.120.000131.) should be cited instead.

Done. We appreciate the suggestion by the reviewer to update the revision about glutamate receptor anion channels with the most recent revision by Stephen F. Traynelis and collaborators.

Reviewer #1 (Remarks to the Author):

THe authors have responded and revised the manuscript well to the comments from the two reviews.They find important determinants and plausible models to understand better the substrate specificity and mechanistic flexibility of the Asc1/CD98hc transport, and they provide a balanced and exciting report of the results.

RESPONSE-TO-REVIEWERS

Reviewer #1 (Remarks to the Author):

THe authors have responded and revised the manuscript well to the comments from the two reviews.They find important determinants and plausible models to understand better the substrate specificity and mechanistic flexibility of the Asc1/CD98hc transport, and they provide a balanced and exciting report of the results.

We thank the reviewer for her/his comments.

We have now adapted the manuscript and figures slightly in respond to the editorial requests.
